# Ossification of the cranium of *Inpaichthys kerri* (Characiformes: Acestrorhamphidae) with discussion of paedomorphic traits in the genus

**DOI:** 10.1111/joa.70052

**Published:** 2025-09-30

**Authors:** Yasmim De Santana Santos, Manoela M. F. Marinho

**Affiliations:** ^1^ Instituto de Biologia Universidade Federal do Mato Grosso do Sul Mato Grosso do Sul Brazil

**Keywords:** neotropics, ontogeny, osteology, sequence of development, tetra puxa‐puxa

## Abstract

The study of development provides valuable information on the evolution of morphological traits, enabling the detection of important evolutionary processes and revealing unique ontogenetic characteristics not observed in adult individuals. The Characiformes is one of the largest groups of Neotropical fish and includes several lineages of small species that have undergone a reduction in body size, a phenomenon often associated with paedomorphosis. In this study, we analyzed an ontogenetic series of a small characiform, *Inpaichthys kerri*, ranging from newly hatched individuals to adults, with the aim of describing in detail the development of the cranial skeleton and establishing its ossification sequence. Seventy‐two skull bones were described, from the first signs of ossification to their adult forms, including the complete sequence of appearance of these structures throughout development. We also identified a unique developmental sequence for the infraorbital bones of *I. kerri*. We highlight the presence of paedomorphic characters found in *I. kerri*, which are shared by its congeners and by other small Characiform lineages, and discuss them based on ontogenetic and phylogenetic information. The information collected and discussed here is unprecedented and is valuable to the understanding of relationships within Acestrorhamphidae.

## Graphical abstract

In this study, we present a detailed description of the cranial development of *Inpaichthys kerri* (Characiformes: Acestrorhamphidae), highlighting important topics such as pedomorphic characters and their relationship with ontogeny and phylogeny, as well as ossification sequences unique to the species.

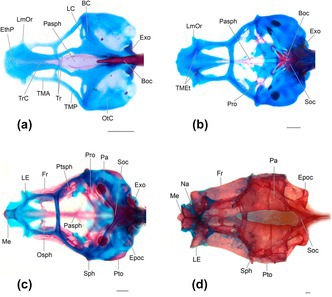

## 1 INTRODUCTION

The embryonic development of the cranium and the establishment of its ossification sequence are key to understanding how reprogramming of developmental mechanisms drives macroevolution, as even minor changes in this structure can significantly impact the adult phenotype (Schoch, 2006). Early developmental stages have long been investigated to uncover heterochronic events and the evolution of morphological traits across vertebrate lineages (e.g., Beriotto et al., 2023; Block & Mabee, 2012; Cubbage & Mabee, 1996; Folly et al., 2022; Marinho, 2022; Mattox et al., 2014).

Within Characiformes, several lineages have undergone evolutionary body size reduction, accompanied by important modifications of the developmental processes (Marinho, 2017, 2022; Mattox et al. 2016). A common consequence of body size reduction is the retention of larval features in adult morphology, a phenomenon known as paedomorphosis (Gould, 1977). This extreme decrease in phylogenetic body size and its associated traits is referred to as miniaturization (Hanken & Wake, 1993). Miniature species are considered those that do not exceed 26 mm in standard length (SL) (Toledo‐Piza et al., 2014; Weitzman & Vari, 1988). Paedomorphic features are common in the morphology of these tiny species, such as poorly developed lateral sensory canals on the head and body, reduction in the number of fin rays and scales, and losses and simplifications of skull bones (Weitzman & Vari, 1988). However, such size limit is arbitrary and recent studies found that paedomorphic features are not solely present in species smaller than 26 mm SL, but non‐miniature species may also undergo developmental truncation and exhibit reductive characters in their morphology as well (Marinho et al., 2024).

In Characiformes, few studies have described skeletal development and provided ossification sequences that enable a comparative analysis to understand the consequences of body size reduction within the order. To date, only five studies are available on this matter: the cranial development of *Brycon moorei* (Bryconidae) (Vandewalle et al., 2005) and *Bario sanctaefilomenae* (Acestrorhamphidae) (Walter, 2012), the complete skeletal development of *Salminus brasiliensis* (Bryconidae) (Mattox et al., 2014), *Makunaima pittieri* (Acestrorhamphidae) (Marinho, 2022), and *Leporinus oliveirai* (Boaretto et al., 2025); and the splanchnocranium development of *Prochilodus argenteus* (Prochilodontidae) (Carvalho & Vari, 2015).

**FIGURE 1:**
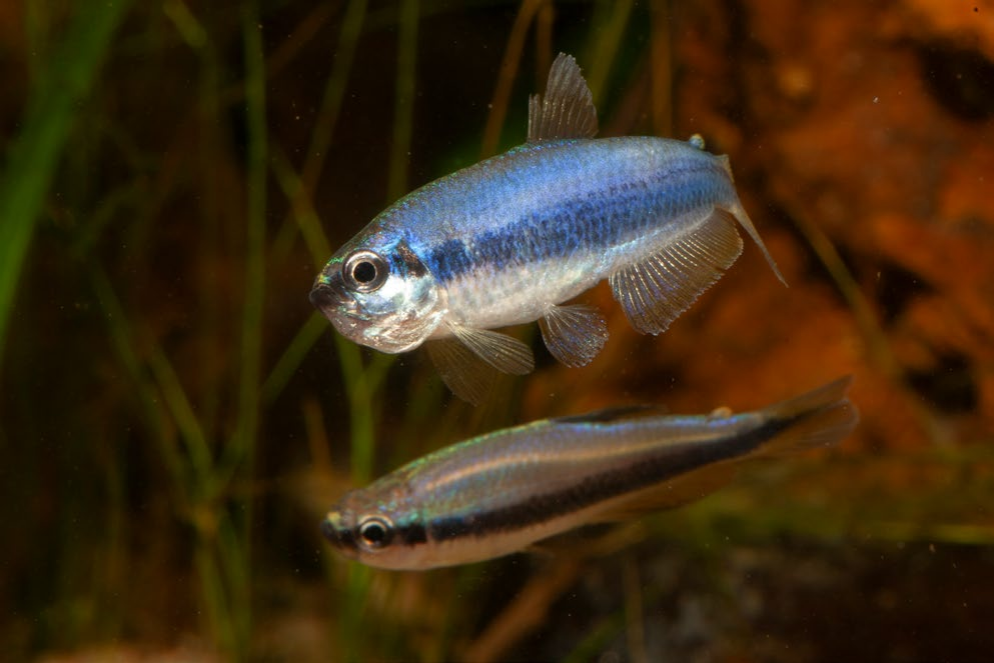
Live specimens of *Inpaichthys kerri*. Male above, female below. Photo by Oliver Lucanus.

*Inpaichthys* Géry and Junk, 1977 (Figure 1) is currently placed in the subfamily Thayeriinae, family Acestrorhampidae, order Characiformes, according to Melo et al. (2024). However, the phylogenetic position of the genus has undergone several revisions in recent years (Ferreira et al., 2024; Melo et al., 2024; Mirande, 2019). Based on phylogenetic analyses combining morphological and molecular data, *Inpaichthys* has been previously assigned to the family Characidae, subfamily Rhoadisinii (Ferreira et al., 2024; Mirande, 2019). More recently, a phylogeny based on ultraconserved elements (UCEs) restricted the family Characidae and elevated large clades traditionally considered subfamilies into family level (Melo et al., 2024), leading to the reclassification of *Inpaichthys*.

The genus *Inpaichthys* currently includes three species: *Inpaichthys kerri* Géry and Junk, 1977, *Inpaichthys nambiquara* (Bertaco & Malabarba, 2007), and *Inpaichthys parauapiranga* Ferreira et al., 2024. Species of *Inpaichthys* are small‐sized fishes, with *I. kerri* reaching the largest size at 28 mm standard length (SL), followed by *I. nambiquara* and *I. parauapiranga*, which reach up to 27 mm and 26.3 mm SL, respectively. Although not considered miniatures, since they exceed 26 mm SL according to the criterion proposed by Weitzman and Vari (1988), *Inpaichthys* species exhibit paedomorphic skeletal features, such as the absence of infraorbitals and the simplification of sensory canals (Ferreira et al., 2024). The extent to which skeletal structures are reduced or simplified as a consequence of body size reduction is not solely determined by adult size but can also be influenced by factors such as the age at which growth ceases and the species growth rate (Hanken & Wake, 1993).

To contribute to the ontogenetic knowledge of the order Characiformes, more specifically of the family Acestrorhamphidae, as well as to their phylogenetic relationships, this article presents a complete description of the development of the cranium of *Inpaichthys kerri*, a non‐miniature species that exhibits a series of paedomorphic skeletal features. We provide the ossification sequence of the cranium of *I. kerri* and discuss its paedomorphic characters in a phylogenetic context.

## 2 MATERIALS AND METHODS

The ontogenetic series of *Inpaichthys kerri* was obtained through captive breeding of adult specimens in an aquarium store. After hatching, the larvae were fed up to 25 days with *Artemia salina* (Linnaeus 1758) and kept in soft, slightly acidic water [hardness 1 dGH (German grade, ∘dH, deutsche Härte), pH 6.8] at temperatures between 23 and 25°C. During the first 15 days, 2–3 larvae were collected every day. After this period, samples were made every 2 days until 55 days after hatching (daf), totallizing 80 larval specimens. The specimens with 55 daf (9.0–9.3 mm SL) were not fully developed; therefore, a further seven adult specimens were purchased from the aquarium store to visualize the fully formed skeleton. Therefore, a total of 87 specimens were analyzed, all deposited at the Zoological collection of the Universidade Federal de Mato Grosso do Sul (ontogenetic series UFMS ZUFMS‐PIS 9287, 80 c&s, 2.6 mm NL–9.3 mm SL; adult specimens UFMS ZUFMS‐PIS 9288, 7 c&s, 26 mm SL–28 mm SL). Specimens were euthanized with eugenol solution (4‐allyl‐2‐methoxyphenol; 0.00005 mL/L) before being fixed in buffered formalin for 24 h. Specimens were then transferred to 70% ethanol and cleared and double‐stained following Taylor and Van Dyke (1985) for visualization of cartilages and bones. For the larval specimens (2.6 mm NL–9.3 mm SL), the following adaptations were made to the Taylor and Van Dyke (1985) method: the larval specimens were immersed in Alcian blue and Alizarin red stain solutions for a maximum of 30 min. During the bleaching stage, the larval specimens were exposed to light and immersed in a solution of distilled water, hydrogen peroxide, and potassium hydroxide for 15 to 20 min. Notochord length (NL) was taken from larvae at the yolk stage and preflexion larval stage, from the anterior tip of the snout to the posterior tip of the notochord. When the first signs of hypurals were seen, standard length (SL) was taken from larvae at flexion, postflexion, and adult stage, from the tip of the snout to the distal end of the third hypural.

In the description, the bones are grouped by anatomical complexes and the common sequence of ossification is given for each region. Since there is a small and expected length interval during which a bone might be present or absent, the length of the smallest specimen in which a bone was first observed is given in the description, along with the length of the specimen in which ossification is always observed from that length on (“fixed presence” sensu Cubbage & Mabee, 1996, Bird & Mabee, 2003).

**FIGURE 2:**
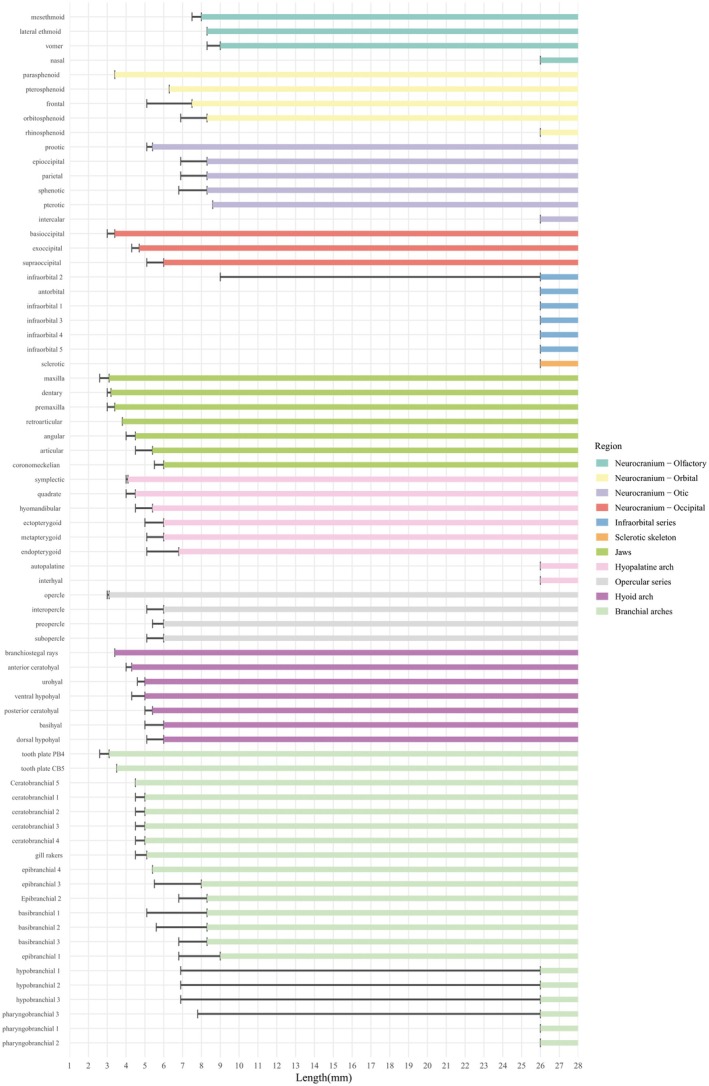
Sequence of ossification of the cranium of *Inpaichthys kerri* organized by anatomical complexes. The gray vertical lines represent the length (NL and SL in mm). Thin horizontal black line represents lengths in which ossification may be present or absent. The colored horizontal bar represents the fixed presence of ossification.

**FIGURE 3:**
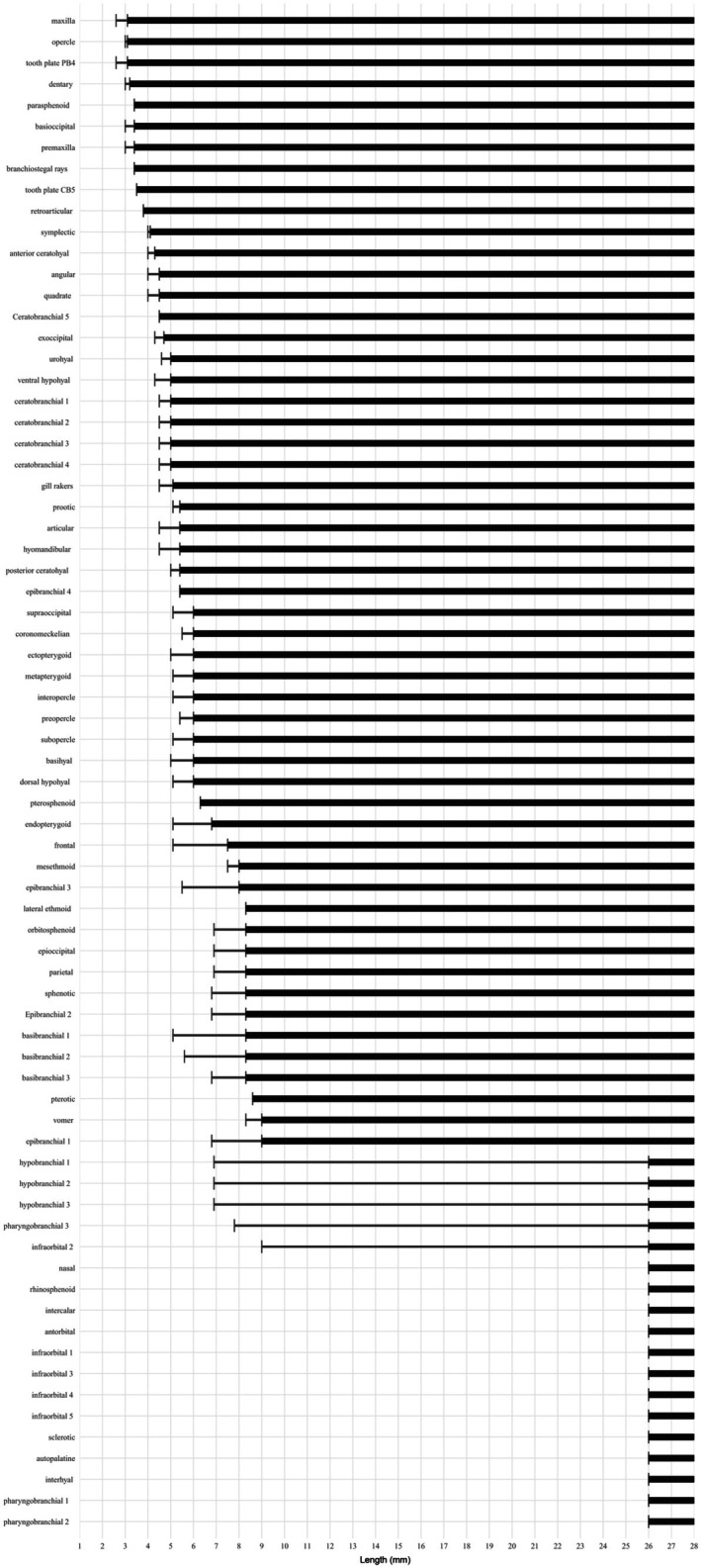
Sequences of ossification of the entire cranium. The gray vertical lines represent the length (NL and SL in mm). Thin horizontal black line represents lengths in which ossification may be present or absent. The black horizontal bar represents the fixed presence of ossification.

The establishment of the sequence of ossification (Figures 2 and 3) is following the method proposed by Cubbage and Mabee (1996), Bird and Mabee (2003), and Mabee et al. (2000). The beginning of ossification was recovered at the first signal of staining with Alizarin. The sequence of development was summarized in two diagrams, one by anatomical regions (Figure 2) and another including the total cranial bones (Figure 3). The bones that were only described in the adult form (antorbital, autopalatine, hypobranchial 1 and 2, infraorbital 1 and 3–5, intercalar, interhyal, nasal, pharyngobranchial 1–3, rhinosphenoid, and sclerotic) are also present in the diagram, but their presence was only recorded from the size of the adult specimens. The terminology of cartilages follows De Beer (1927, 1937), Bertmar (1959), Cubbage and Mabee (1996), Bird and Mabee (2003), and Walter (2012). The terminology of bones follows Weitzman (1962), with some modifications: mesethmoid instead of ethmoid, vomer instead of prevomer, intercalar instead of opisthotic, endopterygoid instead of mesopterygoid or entopterygoid, epioccipital rather than epiotic, anterior ceratohyal instead of ceratohyal, and posterior ceratohyal instead of epihyal (Carvalho et al., 2013; Darlim & Marinho, 2018; Fink & Fink, 1981; Mattox et al., 2014; Patterson, 1975; Vari, 1979, 1995; Zanata & Vari, 2005).

Representative stages of development of anatomical complexes of the specimens in the ontogenetic series were photographed using a Leica M2015A stereomicroscope equipped with a Leica DMC4500 digital camera. The photos were edited with Adobe Photoshop CC 2024. Information on reductive characters in the genus *Inpaichthys* and relatives was compiled from the matrix of Ferreira et al. (2024).

## 3 RESULTS

*Inpaichthys kerri* has 72 bones in the cranial region, considering each serial bone (e.g. ceratobranchials 1–5) as a single ossification. Below we describe the development of each bone of the neuro‐and splanchnocranium by region.

### 3.1 Olfactory region (Figures 4, 5, 6)

**FIGURE 4:**
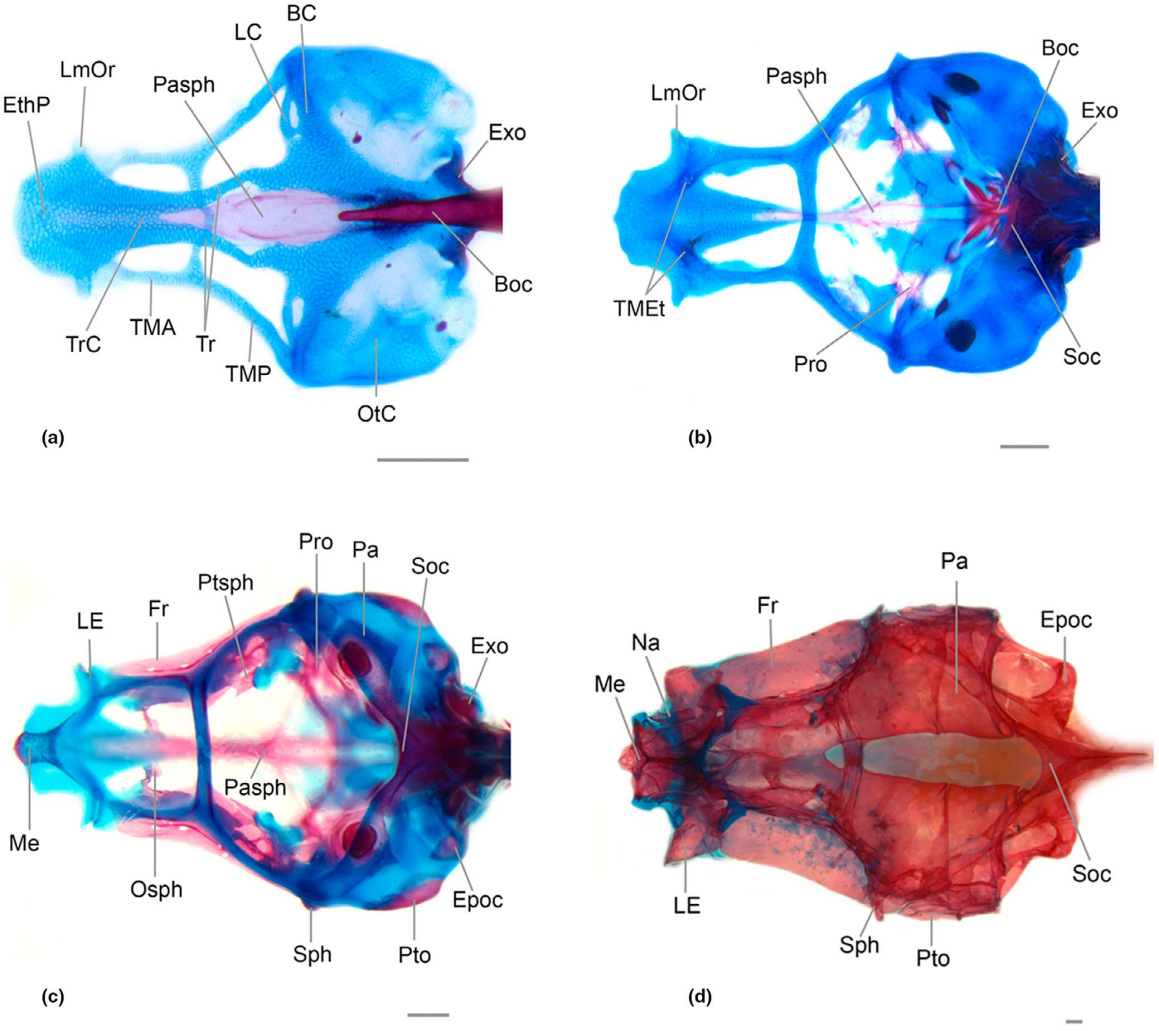
Neurocranium of *Inpaichthys kerri*, dorsal view. (a) 4.5 mm NL. (b) 7.5 mm SL. (c) 9.3 mm SL. (d) 26 mm. BC, basicapsular commissure; Epoc, epioccipital; EthP, ethmoid plate; Exo, exoccipital; Fr, frontal; LC, lateral commissure; LE, lateral ethmoid; LmOr, *lamina orbitonasalis*; Me, mesethmoid; Na, nasal; Osph, orbitosphenoid; OtC, otic capsule; Pa, parietal; Pasph, parasphenoid; Pro, prootic; Pto, pterotic; Ptsph, pterosphenoid; Soc, supraorbital; Sph, sphenotic; TMA, *taenia marginalis* anterior; TMEt, *taenia marginalis ethmoidalis*; TMP, *taenia marginalis* posterior; Tr, *trabecula*; TrC, *trabecula communis*. Scale bar 0.2 mm.

Common sequence of ossification: mesethmoid – lateral ethmoid – vomer – nasal.

#### 3.1.1 Mesethmoid

The mesethmoid starts to ossify as a thin splint of bone at the anterior margin of the ethmoid plate cartilage, between the ascending process of the premaxilla at 7.5 mm SL, with fixed presence at 8.0 mm SL. At 8.6 mm SL, the bone is large posteriorly, with a rectangular shape. A very small bifurcation can be seen anterior to the region where the *orbitonasalis* and *taenia marginalis ethmoidalis* cartilages meet. At 9.3 mm SL, the posterior bifurcation is long (Figure 4c). In the adult form, the mesethmoid is broad and approximately rhomboidal, with a short anterior projection between the contralateral ascending processes of the premaxilla. The posterior end lacks any bifurcation and is situated between the two frontals. In most adult specimens, the lateral wings are well‐developed, but there is also a less developed condition surrounded by a large portion of cartilage. Ventral divergent lamella is present.

#### 3.1.2 Lateral ethmoid

**FIGURE 5:**
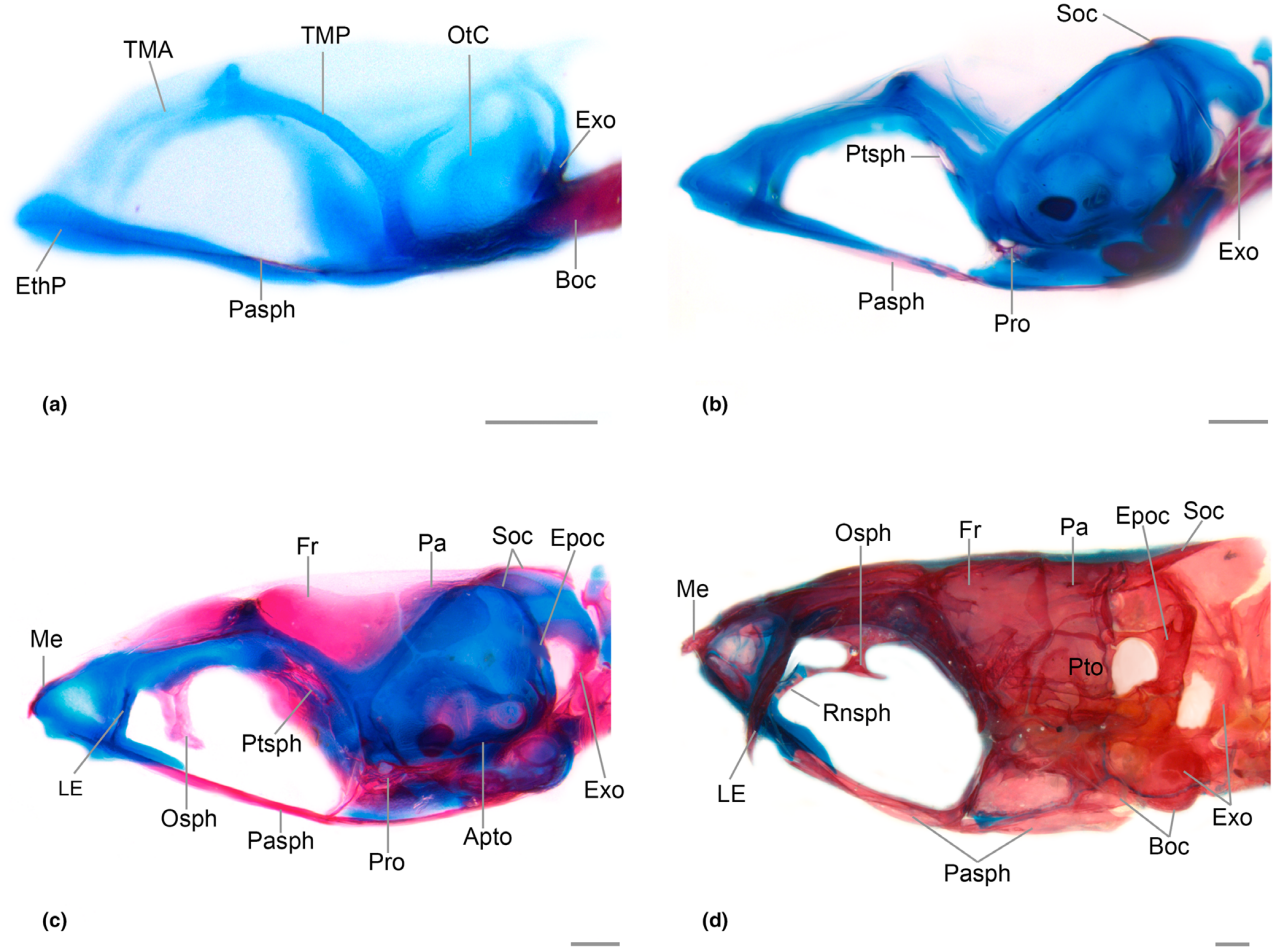
Neurocranium of *Inpaichthys kerri*, lateral view. (a) 4.5 mm NL. (b) 7.5 mm SL. (c) 9.3 mm SL. (d) 26 mm SL. Apto, autopterotic; Epoc, epioccipital; EthP, ethmoid plate; Exo, exoccipital; Fr, frontal; LE, lateral ethmoid; Me, mesethmoid; Osph, orbitosphenoid; OtC, otic capsule; Pa, parietal; Pasph, parasphenoid; Pro, prootic; Pto, pterotic; Ptsph, pterosphenoid; Rnsph, rhinosphenoid; Soc, Supraorbital; TMA, *taenia marginalis anterior*; TMP, *taenia marginalis posterior*. Scale bar 0.2 mm.

**FIGURE 6:**
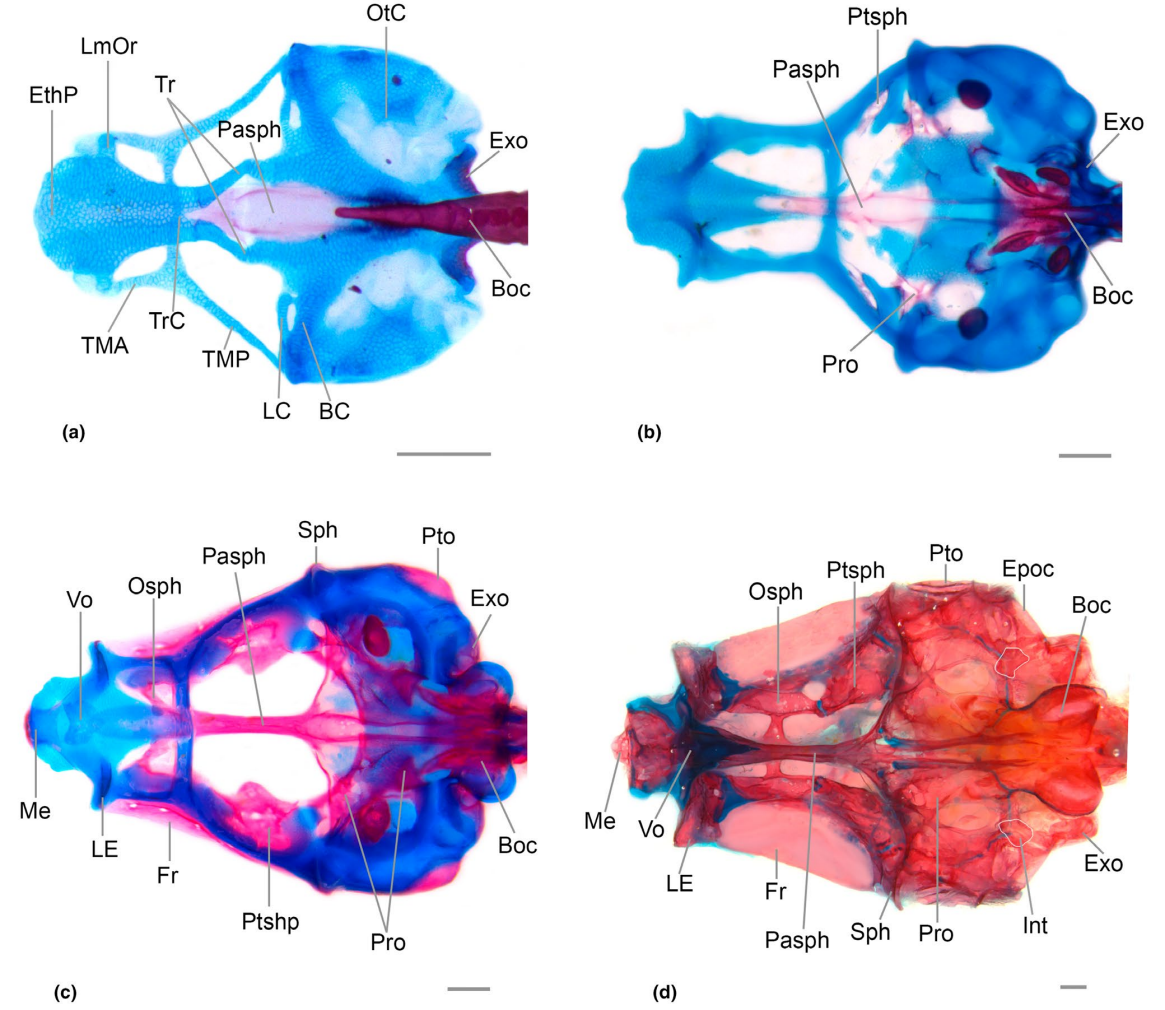
Neurocranium of *Inpaichthys kerri*, ventral view. (a) 4.5 mm NL. (b) 7.5 mm SL. (c) 9.3 mm SL. (d) 26.3 mm SL. BC, *basicapsular commissure*; Epoc, epioccipital; EthP, ethmoid plate; Exo, Exoccipital; Fr, frontal; Int, intercalar; LC, *lateral commissure*; LE, lateral ethmoid; LmOr, *lamina orbitonasalis*; Me, mesethmoid; Osph, orbitosphenoid; OtC, otic capsule; Pasph, parasphenoid; Pro, prootic; Pto, pterotic; Ptsph, pterosphenoid; Soc, Supraorbital; Sph, sphenotic; TMA, *taenia marginalis anterior*; TMEt, *taenia marginalis ethmoidalis*; TMP, *taenia marginalis posterior*; Tr, trabecula; TrC, *trabecula communis*; Vo, vomer. Scale bar 0.2 mm. *Trabeculae are folded in figure A.

The lateral ethmoid is a paired bone that appears as a perichondral ossification in the lateral margin of the *lamina orbitonasalis* at 8.3 mm SL. At 9.1 mm SL, the bone replaces the cartilage medially and ventrally, and surpasses the lateral margin of the cartilage, almost reaching its ventral tip (Figures 4, 5, 6). In adult specimens, the vertical portion of the lateral ethmoid has replaced almost the entire cartilage of the *lamina orbitonasalis*, reaching the frontal and orbitosphenoid dorsally and surpassing the parasphenoid ventrally (Figure 5d). In adults, there is an anterior process, slender and short, distant from its counterpart and not reaching the vomer medially. There is broad space between the anterior process of the lateral ethmoid and the ventral divergent lamella of the mesethmoid.

#### 3.1.3 Vomer

It is a dermal bone that begins to ossify ventrally to the anterior portion of the ethmoid plate at 8.3 mm SL; its presence is fixed at 9.0 mm SL. At 9.1 mm SL, the posterior portion of the vomer is ossified and contacts the parasphenoid posteriorly. At this stage, it is a heart‐shaped bone (Figure 6c). In the adult, the vomer has a similar shape to the anterior stage, but with a lamellar expansion posteriorly that is much narrower than the anterior region and is approximately triangular in shape, reaching the anterior half of the posterior projection of the ethmoid cartilage. The anterior region of the vomer is restricted to the center of the ethmoid cartilage.

#### 3.1.4 Nasal

The nasal could only be observed in the adult, as the oldest larval specimen analyzed (9.3 mm SL) does not have it. In adults, the nasal is restricted to the tubular sensory canal, which is the anterior portion of the supraorbital canal, lacking any bony lamellae. It is thin and slightly concave (Figure 4d), situated between the anterior tip of the frontal and the dorsal tip of the ascending process of the premaxillary toward the lateral wing of the mesethmoid.

### 3.2 Orbital region (Figures 4, 5, 6)

Common sequence of ossification: parasphenoid – pterosphenoid – frontal – orbitosphenoid – rhinosphenoid.

#### 3.2.1 Parasphenoid

Ossification starts at 3.4 mm NL in the hypophyseal fenestra, between the *trabeculae*, as a unique lamellar structure, narrow and elongate, that contacts the *trabeculae communis* anteriorly and the anterior tip of the basioccipital posteriorly (Figures 4 and 6). By 4.1 mm NL, there are two parallel ventrolateral thin splints of bone that extend posteriorly, surpassing the tip of the basioccipital. At 6.8 mm SL, the parasphenoid is expanded anteriorly at the medial portion of the *trabeculae communis*. At this stage, the lateral wings can be seen as two small projections on either side of the bone, directed toward the anterior portion of the prootic (Figure 6b). At 9.1 mm SL, the anterior portion of the parasphenoid is in contact with the vomer, the posterior portion has a deep bifurcation that reaches the medial region of the basioccipital, and its lateral wings are connected to the anterior portion of the prootic (Figure 6c). There are no significant changes in the adult form, except a deep ventral curvature.

#### 3.2.2 Pterosphenoid

The pterosphenoid begins to ossify perichondrally at 6.3 mm SL as a thin lamella posterior to the epiphyseal bar, in the ventral margin of the *taenia marginalis*. It spreads ventrally and medially, and at 9.3 mm SL, it is approximately oval, with a small foramen at its center (Figure 4c). In the adult form, the bone has an approximately rectangular shape. Anterodorsally, at the articulation with the orbitosphenoid, there is an oval foramen bordered dorsally by the frontal. Posteriorly, the bone articulates with the prootic.

#### 3.2.3 Frontal

It appears at 6.3 mm SL (fixed presence at 8.0 mm SL) as a thin splint of dermal ossification lateral to the *taenia marginalis*, above the orbit. At 8.6 mm SL, it is a lamellar bone that extends from the lateral ethmoid, falling short of the parietal posteriorly. At this stage, it almost reaches its counterpart at the epiphyseal bar. By 9.3 mm SL, it contacts the parietal posteriorly and there is a lateral flap above the orbit (Figure 4c). At this stage, there is a small bony wall above the orbit, which is the first sign of the ossification around the supraorbital canal in formation. In adults, the frontals contact their counterpart anteriorly, leaving only a small fontanel anterior to the epiphyseal bar. The anterior end reaches the posterior opening of the nasal and has a “V” shape, where the posterior region of the mesethmoid fits in. The posterior end overlaps the parietal. In the adult condition, the supraorbital canal extends to the nasal anteriorly and its frontal portion reaches the posterior region of the epiphyseal bar ending in the tubule SO7, without the presence of the parietal branch or tubule SO8 (Figure 5d).

#### 3.2.4 Orbitosphenoid

The orbitosphenoid first appears at 6.9 mm SL in the ventral margin of the *taena marginalis*, anterior to the epiphyseal bar (fixed presence at 8.6 mm SL). At 8.6 mm SL, the bone is much expanded ventrally toward the parasphenoid and at 9.3 mm SL this expansion contacts its counterpart (Figure 5b,c). In adults, the orbitosphenoid is relatively small and slender; it articulates synchondrally with the lateral ethmoid anterodorsally and meets the pterosphenoid posteriorly. The orbitosphenoid does not reach the parasphenoid ventrally (Figure 5d).

#### 3.2.5 Rhinosphenoid

The ossification could only be observed in the adults as it is not present at the oldest specimen analyzed (9.3 mm SL). In adults, it is poorly developed, small, and narrow, with concave sides. In the specimen in Figure 5d, ossification has not replaced all the cartilage, leaving the bone with this appearance. Dorsally, it has no expansion and connects with the orbitosphenoid by means of a piece of cartilage; ventrally, it connects with the *trabecula communis*, not reaching the parasphenoid (Figure 5d).

### 3.3 Otic region (Figures 4, 5, 6)

Common sequence of ossification: prootic – (sphenotic + parietal + epioccipital) – pterotic.

#### 3.3.1 Prootic

The prootic begins to ossify and is consistently present at 5.4 mm SL. The ossification starts perichondrally in the *lateral commissure* and *basicapsular commissure* cartilages that surround the *trigemino‐facialis* foramen (Figure 6a,b). By 7.5 mm SL, the prootic has expanded considerably posteriorly and medially toward the parasphenoid. It is possible to distinguish centers of ossification more apparent around the auditory foramen, anterior to the saccular capsule, and lateral to the parasphenoid (Figure 6b). At 9.1 mm SL, the prootic is larger and more evident, almost connected to the exoccipital posteriorly. In the adult, the bone is well‐developed; the ventral portion has a projection that connects with the posterior region of the parasphenoid and posteriorly connects with the exoccipital and pterotic.

#### 3.3.2 Sphenotic

Ossification starts in the process postorbital, located at the anterolateral tip of the otic capsule at 6.8 mm SL, with fixed presence at 8.3 mm SL. The sphenotic spine appears at 9.1 mm SL as a small projection (Figures 4c and 6c), and at 9.3 mm SL, it is larger, growing toward the frontal and pterotic. In adults, the sphenotic is partially covered dorsally by the frontal, articulated by a patch of cartilage with the pterosphenoid anteriorly and with the pterotic posteriorly. The spine is slender and short, its distal end aligned with the dorsal margin of the infraorbital 4.

#### 3.3.3 Parietal

The dermal bone appears at 6.9 mm SL, with fixed presence at 8.3 mm SL, at a membrane situated laterally at the posterodorsal region of the neurocranium, anterior to the *tectum synoptic* cartilage. At 9.3 mm SL, it is approximately rectangular, contacting the pterotic ventrally, the supraoccipital posteriorly, and the frontal anteriorly (Figure 4c). In adults, the parietal counterparts have no contact, leaving a wide parietal fontanel. The posterior margin of the parietal is traversed by the supratemporal canal.

#### 3.3.4 Epioccipital

The epioccipital begins to ossify perichondrally along the median portion of the cartilage that covers the vertical semicircular canal of the otic capsule at 6.9 mm SL with fixed presence at 8.3 mm SL. At 9.1 mm SL it is possible to see a slightly circular ossification at the same region (Figure 5c). Ossification spreads dorsally and ventrally and in the adult form, the main body of the epioccipital articulates with the supraoccipital dorsally and the exoccipital ventrally. The bridge of the epioccipital projects from the middle portion of the bone, crossing the posttemporal fossa and connecting anteriorly with the pterotic and parietal.

#### 3.3.5 Pterotic

The pterotic has two types of ossification, perichondral (autopterotic) and dermal (dermopterotic) (Marinho, 2022). The first sign of ossification seen in *Inpaichthys kerri* is the autopterotic, at the posterolateral region of the cartilage surrounding the horizontal semicircular canal of the otic capsule, at 8.6 mm SL (Figure 5c). It was not possible to observe the formation of the dermopterotic, which is seen only in adults. In adult specimens, the bone is well‐developed, the autopterotic covers the entire horizontal semicircular canal, having a cylindrical and slightly convex shape, with a small thorn from which the tendon from the protactor pectoralis muscle originates. The dermopterotic is limited dorsally by the frontal and parietal and has the otic canal running horizontally along its length.

#### 3.3.6 Intercalar

The intercalar could only be seen in the adult specimens (Figure 6d). It has an approximately rectangular shape and is located in the articulation region between the pterotic and prootic. The bone has a tendon that connects it to the posttemporal bone of the pectoral fin, but the end of the tendon connected to the posttemporal bone is not ossified.

### 3.4 Occipital region (Figures 4, 5, 6)

Common sequence of ossification: Basioccipital – Exoccipital – Supraoccipital.

#### 3.4.1 Basioccipital

The basioccipital appears early in development, at 3.0 mm NL, with a fixed presence at 3.4 mm SL. The bone appears as a perichondral ossification surrounding the anterior portion of the notochord (Figures 4a and 6a). By 4.5 mm NL, the basioccipital reaches the parachordal cartilage laterally, and at 5.1 mm SL, the region anterior to the tip of the notochord is more ossified. At 9.3 mm SL, the expansion of the bone forms a “V”‐shaped ossification anterior to the tip of the notochord. At this stage, the bone also reaches the ventral surface of the saccular capsule laterally and covers part of the ventral portion of the lagenar capsule. In the adult, the basioccipital covers the entire ventral surface of the lagenar capsule and reaches the prootic anteriorly. It also has a ventral longitudinal lamella that reaches the posterior edge of the bone.

#### 3.4.2 Exoccipital

Ossification begins in the medial portion of the occipital arch at 4.3 mm NL with fixed presence at 4.7 mm NL (Figures 4a and 6a). At 6.8 mm SL, the ossification covers almost the entire occipital arch, except for a small region more ventral connected with basioccipital. At 7.5 mm SL, the ossification surrounds the occipital foramen (Figure 6b). At 9.3 mm SL, the posterior portion surrounding the occipital foramen transforms into a bridge anterior to the schapium (Figure 5c). At this point, the bone covers most of the dorsal portion of the lagenar capsule. In adults, the exoccipital completely covers the dorsal surface of the lagenar capsule, articulates with the basioccipital ventrally, and forms the posterior border of the occipital foramen, reaching dorsally to the ventral region of the supraoccipital.

#### 3.4.3 Supraoccipital

The supraoccipital starts to ossify at 6.0 mm SL, with a fixed presence from the same size. The ossification begins at the anterior margin of the *synoptic tectum* (Figure 4b). At 7.5 mm SL, the bone has grown posteriorly. At 8.6 mm SL, the ossification is expanded ventrally in the direction of the exoccipital, and there is a posterodorsal extension that later will form the supraoccipital spine. In adult specimens, the supraoccipital is V‐shaped in dorsal view, with its posterior end formed by the supraoccipital spine that reaches the anterior region of the dorsal end of the neural complex of Weber's apparatus. Ventrally, it connects with the epioccipital laterally and the exoccipital medially.

### 3.5 Infraorbital series (Figure 7)

**FIGURE 7:**
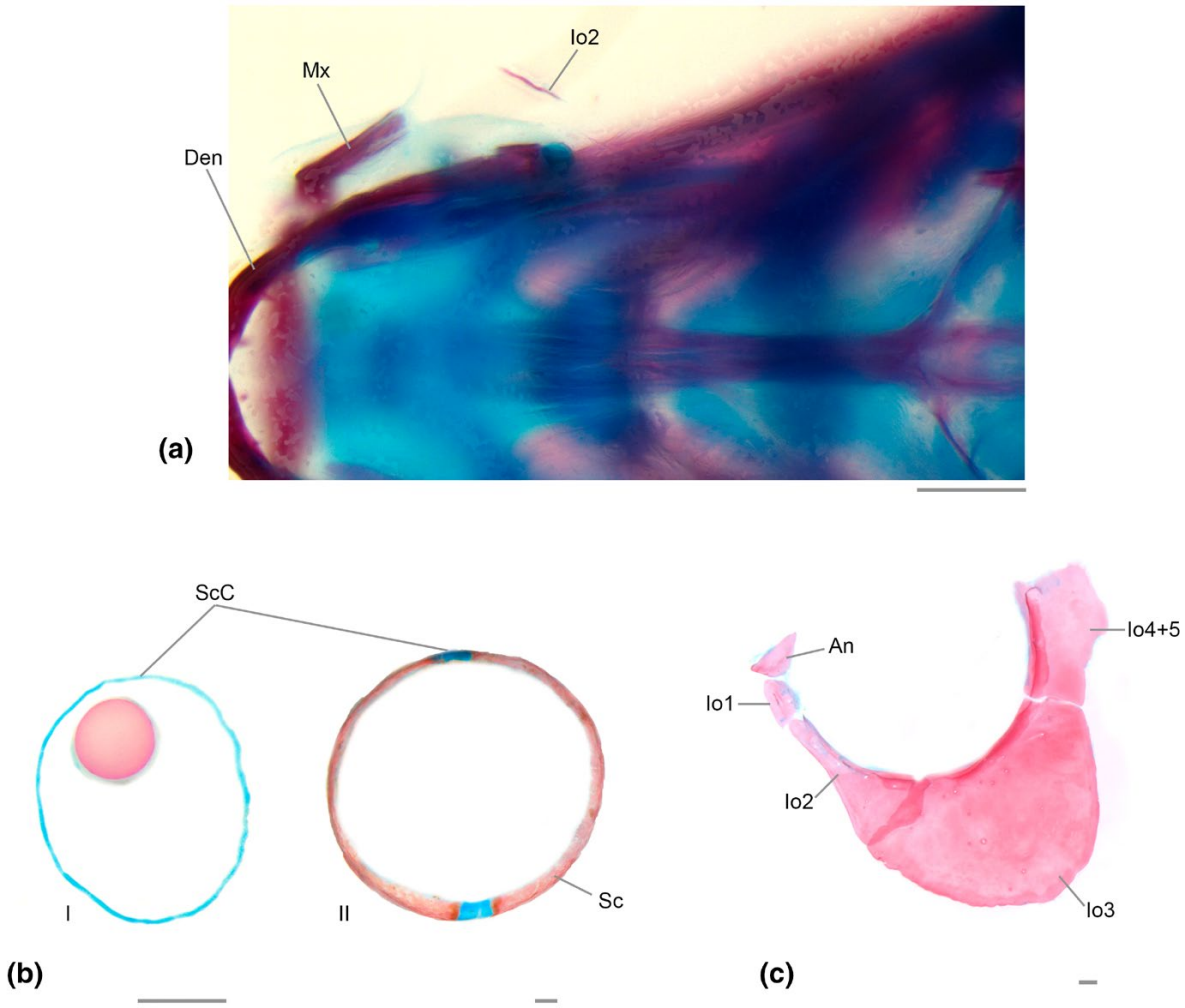
Infraorbitals and sclerotic of *Inpaichthys kerri*. (a) anterior portion of neurocranium, dorsal view, 9.0 mm SL. (b) Sclerotics, I, 5.1 mm SL; II, 28.0 mm SL. (c) Infraorbital series of adult specimen. An, antorbital; Io1‐5, infraorbitals; Mx, maxilla; Sc, sclerotic; ScC, sclerotic cartilage. Scale bar 0.2 mm.

Common sequence of ossification: Infraorbital 2– (Antorbital + Infraorbital 1 + Infraorbital 3–5).

**FIGURE 8:**
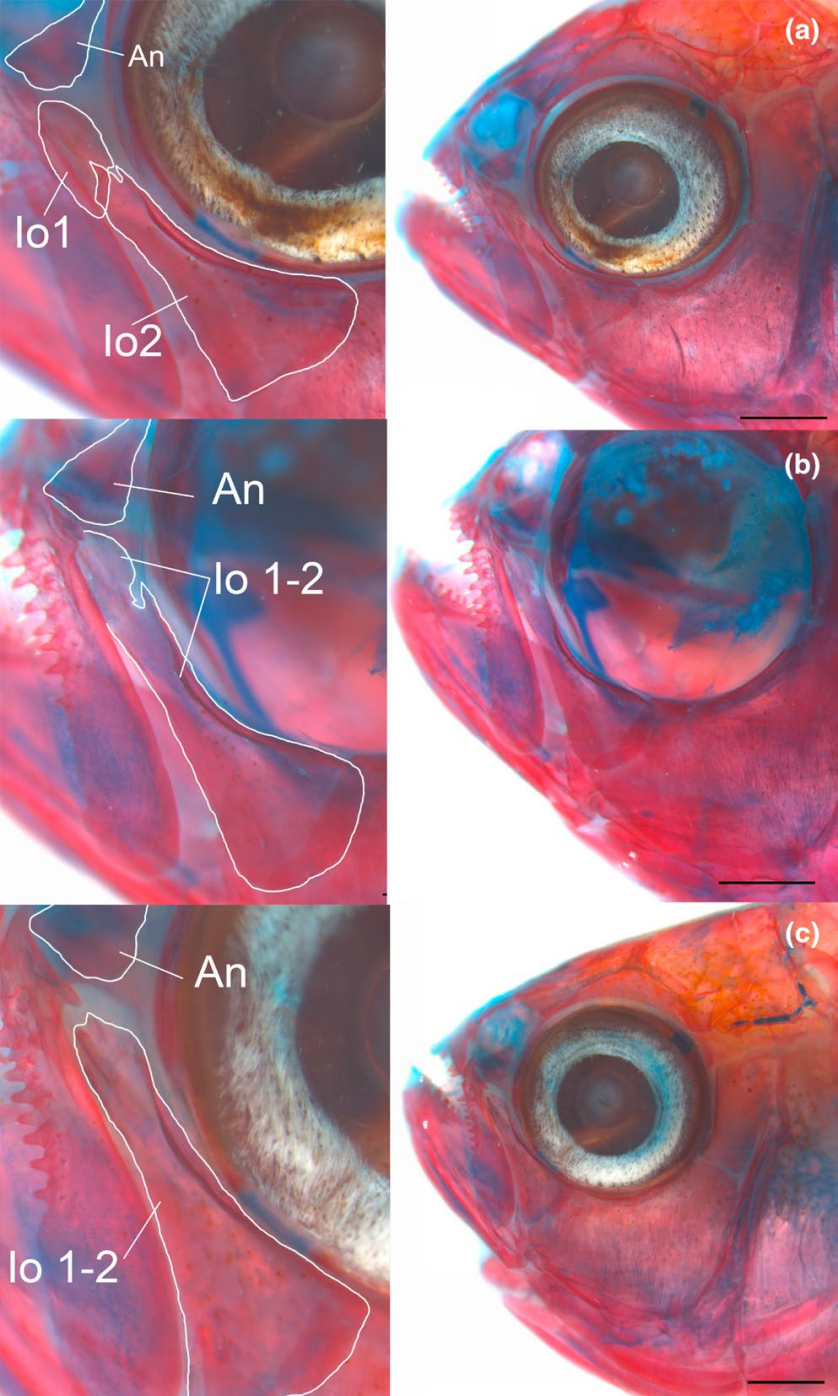
Variation of infraorbital 1 and 2 in adult specimens of *Inpaichthys kerri* (all with 28 mm SL). (a) Infraorbital 1 and 2 separated. (b) Infraorbital 1 and 2 partially fused. (c) Infraorbital 1 and 2 fused. An, antorbital; Io 1–2, infraorbitals. Scale bar 1 mm.

The infraorbital series starts with the formation of infraorbital 2 at 9.0 mm SL, which ossifies anteroventrally in the neurocranium, posterior to the maxilla and dorsal to the anguloarticular, as a thin splinter of bone positioned horizontally (Figure 7a). It was not possible to determine the sequence of ossification of the other components of the infraorbital series, as the oldest larval specimen only has infraorbital 2. In the adult specimen, the infraorbital series is composed of the antorbital and infraorbitals 1, 2, 3 and 4 + 5 (see Discussion). The antorbital is triangular in shape and does not overlap with any other bone. The condition of infraorbital 1 is variable in the seven adult specimens of *Inpaichthys kerri* examined, presenting three distinct character states: infraorbital 1 separated from infraorbital 2 (Figure 8a) (*n* = 4 specimens), infraorbital 1 partially fused with infraorbital 2 (Figure 8b) (*n* = 1 specimen) or infraorbital 1 totally fused with infraorbital 2 (Figure 8c) (*n* = 2 specimens). When present and autogenous, infraorbital 1 is small, poorly developed and has no sensory canal. When autogenous, infraorbital 2 is elongated and narrow, with a slightly wider posterior end. When fused, infraorbital 1 and 2 are elongated, occupying the same position as autogenous infraorbitals 1 and 2 in the other specimens, almost the same size as the maxilla, separated anteriorly from the antorbital and with the posterior end overlapping the infraorbital 3. Infraorbital 3 is approximately semicircular in shape. Infraorbital 4 + 5 is rectangular, vertically elongated, with the dorsal end at the horizontal through the dorsal end of the hyomandibular and operculum. The infraorbital sensory canal runs along infraorbitals 2 to 4 + 5 at their entire orbital margin, except infraorbital 2, in which the canal is only present at its distal orbital end.

### 3.6 Sclerotic bones (Figure 7)

#### 3.6.1 Sclerotic

Ossification of the sclerotic was only seen in adult specimens, with two ossification centers, one anterior and one posterior, interspersed by two patches of cartilage.

### 3.7 Jaws (Figure 9)

**FIGURE 9:**
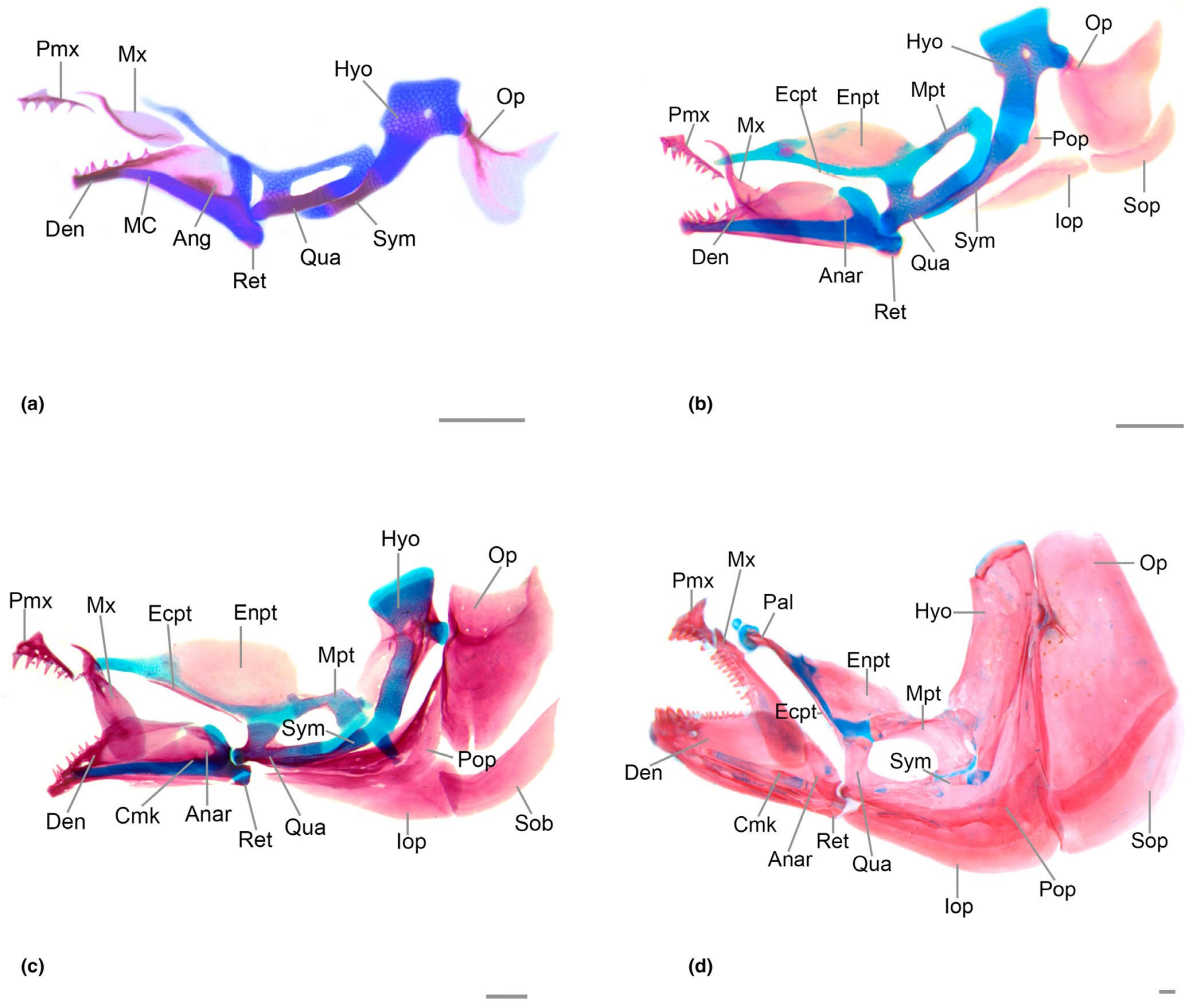
Jaws, hyopalatine arch and opercular series of *Inpaichthys kerri*. (a) 4.5 mm NL. (b) 5.1 mm SL. (c) 9.3 mm SL. (d) 28 mm SL. Anar, anguloarticular; Ang, angular; Cmk, coronomeckelian; Den, dentary; Ecpt, ectopterygoid; Enpt, endopterygoid; Hyo, hyomandibular; Iop, interopercle; Mx, maxilla; Mpt, metapterygoid; Op, opercle; Pal, palatine; Pmx, premaxilla; Pop, preopercle; Qua, quadrate; Ret, retroarticular; Sob, subopercle; Sym, sympletic. Scale bar, 0.2 mm.

Common sequence of ossification: maxilla – dentary – premaxilla – retroarticular – angular – articular – coronomeckelian.

#### 3.7.1 Maxilla

**FIGURE 10:**
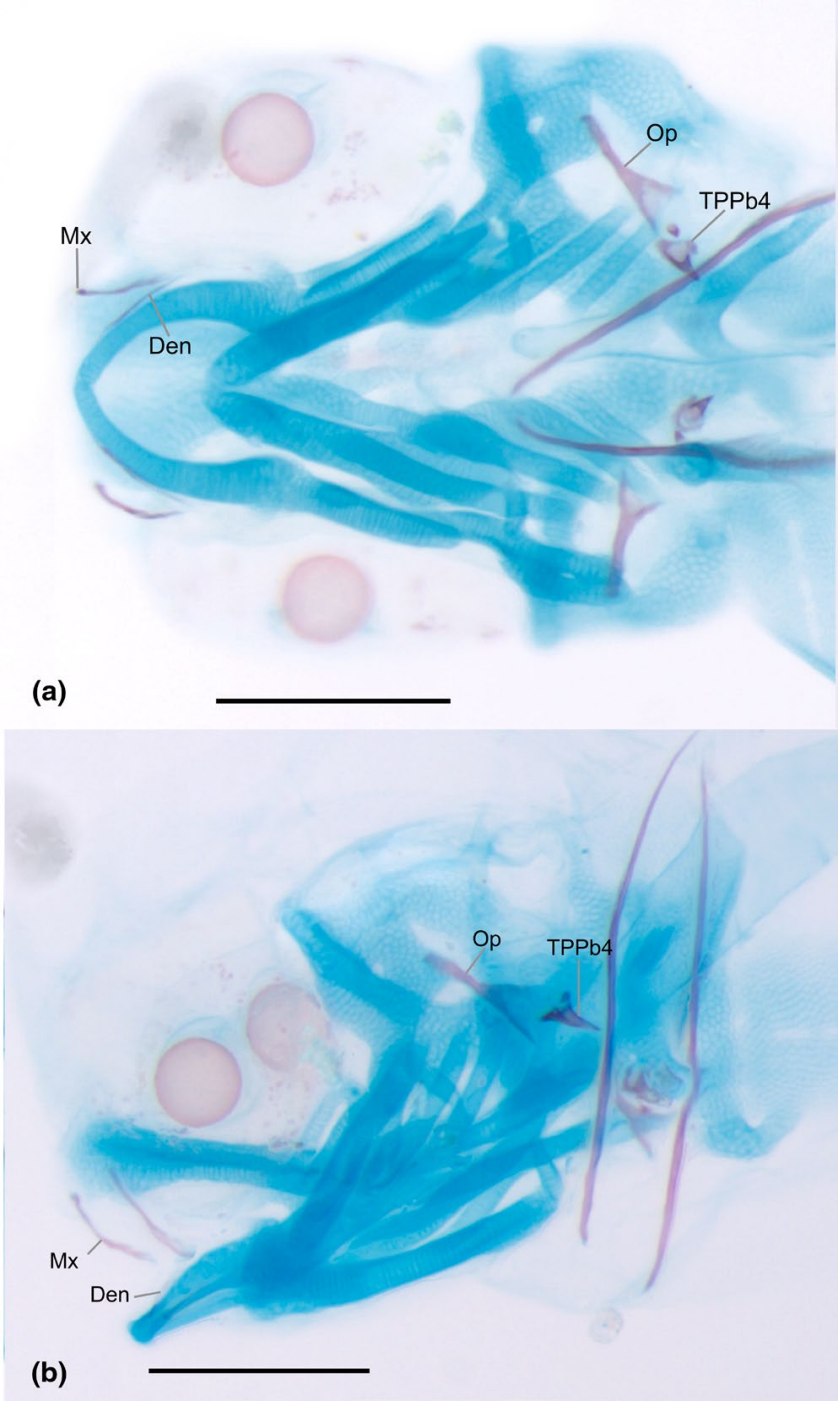
First ossifications of the cranium of *Inpaichthys kerri* of 3.2 mm NL. (a) Ventral view. (b) Lateral view. Den, dentary; Mx, maxilla; Op, opercle; TPPb4, tooth plate of pharyngobranchial 4. Scale bar 0.2 mm.

The maxilla is one of the first bones to appear, at 2.6 mm NL as a thin dermal splint of bone positioned laterally, dorsal to the Meckel's cartilage and below the ethmoid plate (Figure 10). At 4.2 mm NL, the anterior tip of the maxilla is curved toward the premaxilla, while the posterior end becomes wider with a dorsal, flattened lamellar expansion. At 5.1 mm SL, it is elongated, with its anterior portion thin and curved and its posterior portion enlarged with rounded edges (Figure 9b). At this stage, the maxilla is edentulous. Only after 8 mm SL do two small and conical teeth emerge. At 8.6 mm SL, the anterior tip reaches the middle of the premaxilla. In adults, the ascending process of the maxilla is pointed and directed to the medial portion of the premaxilla. The anterior portion of the maxilla has a straight margin, with six to ten teeth at its dorsal half. Overall, the two anteriormost teeth are larger, with one to five cuspids, followed by smaller conical teeth. There is a presence of a tubule for the passage of blood vessels on the dorsal margin of the maxilla. The posterior end of the maxilla is aligned with the posterior end of infraorbital 2.

#### 3.7.2 Dentary

The dentary appears as a thin and elongated bone, horizontally positioned over the anterior portion of Meckel's cartilage, with a lamellar posterodorsal end slightly wider than the rest of the bone (3 mm NL) (Figure 10). The first two conical teeth appear at 4.2 mm NL. At 5.1 mm SL, the ventral margin of the dentary is more conspicuous, present horizontally below the Meckel's cartilage. At this stage, the lamellar part grows toward the coronoid process and the number of teeth increases considerably to 11. A small foramen appears at 8.6 mm SL, at the anterior portion of the dentary, dorsal to the anterior region of Meckel's cartilage (Figure 9c). The number of teeth remains almost the same up to 9.3 mm SL. At this stage, the lamellar posterodorsal region bypasses the dorsal end of the coronoid process and contacts the anguloarticular, and the ventral margin almost reaches the retroarticular posteriorly. In the adult form, the dentary has four large, tricuspid anterior teeth followed by 10 smaller, conical teeth. The mandibular sensory canal is present in the lateroventral region of the dentary.

#### 3.7.3 Premaxilla

The premaxilla appears as a thin splint of bone, situated anterior to the ethmoid plate. The smallest individual analyzed already presents one conical tooth positioned medially at the premaxilla (3.4 mm NL) (Figure 9a). Ossification progresses laterally toward the maxilla. At 4.5 mm NL, there are 4–5 tiny conical teeth. At this stage, the ascending process of the premaxilla appears, and the lateral portion of the premaxilla reaches the maxilla. The ascending process is more pronounced at 7.5 mm SL, reaching the mesethmoid at 9.1 mm SL. In the adult specimens, the premaxilla has one tooth row with 4–5 teeth, with one to three cusps. The ascending process is triangular and reaches the mesethmoid dorsally.

#### 3.7.4 Retroarticular

The ossification of retroarticular begins at 3.8 mm NL in the posterior end of Meckel's cartilage, in the region where the interoperculo‐mandibular ligament attaches (Figure 9a). As the bone develops, it becomes triangular in shape, and only at 9.1 mm SL does its anterior ventral tip reach the ventral part of the dentary. In adults, it is a small triangular bone that connects with the dentary anteriorly and with the anguloarticular dorsally.

#### 3.7.5 Anguloarticular

The anguloarticular is a compound bone, formed by a dermal angular and an endochondral articular. The first ossification to start is the angular, which ossifies dorsal to the posterior portion of Meckel's cartilage, anterior to the coronoid process (4.3 mm SL, with fixed presence at 4.5 mm SL) (Figure 9a). The articular ossifies later, appearing for the first time at 4.5 mm SL, with consistent presence at 5.5 mm SL, in the posterior region of Meckel's cartilage, dorsal to the retroarticular, near the region of articulation with the quadrate (Figure 9b). From 6 mm SL, the angular and articular are fused in all specimens, forming a single bone. At 8.6 mm SL, the anguloarticular expands ventrally, contacting the ventral portion of the dentary and almost reaching the retroarticular. Posteriorly, the bone covers Meckel's cartilage laterally, with the exception of the coronoid process. In adults, the anguloarticular is limited anteriorly by the dentary and articulates posteriorly with the quadrate. The mandibular canal is not present in the anguloarticular.

#### 3.7.6 Coromeckelian

The coronomeckelian is a small bone that first appears at 5.5 mm SL, but its presence is fixed at 6.0 mm SL. The coronomeckelian ossifies in the posterior portion of Meckel's cartilage, from the tip of the Meckel tendon that attaches in this region. Initially, the bone has a form of a small rounded grain, and at 9.3 mm SL (Figure 9c), it progresses to a roughly rectangular shape. In adults, the coronomeckelian is more evident, but its shape does not change considerably.

### 3.8 Hyopalatine arch (Figure 9)

Common sequence of ossification: symplectic– quadrate– hyomandibular– (metapterygoid + ectopterygoid)– endopterygoid.

#### 3.8.1 Sympletic

The sympletic starts to ossify perichondrally in the ventral tip of the hyosymplectic cartilage (at 4.0 mm NL with fixed presence from 4.1 mm NL) (Figure 9a). The development of the sympletic is simple, compared with other bones; its shape does not change considerably throughout the ontogeny. Ossification progresses anteriorly and posteriorly. By 9.3 mm SL, the bone reaches almost the same length as the metapterygoid‐quadrate fenestra (Figure 9c). In adults, the sympletic has two cartilaginous ends: the anterior one in contact with the anteriormost region of the quadrate and the posterior one separated from the ventral region of the hyomandibular by a patch of cartilage (Figure 9d). The bone has a thin, elongated shape, with the anterior region being slightly thinner than the rest of the bone.

#### 3.8.2 Quadrate

The quadrate appears early in the development at 4.0 mm NL, located in the ventral margin of palatoquadrate cartilage, posterior to the articular process that connects with the anguloarticular (fixed presence at 4.5 mm NL) (Figure 9a). From this point, the bone progresses in two different directions. At 4.5 mm NL, it expands dorsally toward the ventral margin of the endopterygoid and expands posteriorly toward the hyomandibular, forming an “L” shape (Figure 9b). A small lamellar portion emerges at 6.8 mm SL in the anteroventral portion of the metapterygoid‐quadrate fenestra and grows toward the center of the fenestra. At 9.1 mm SL, the bone covers the articular process, contacts the sympletic posteriorly, and almost reaches the endopterygoid dorsally (Figure 9c). In adults, the quadrate takes on an L‐shape, with the anterodorsal region being much wider than the posteroventral region. The anterodorsal end contacts the ectopterygoid directly and is separated from the endopterygoid and metapterygoid by a piece of cartilage. The posteroventral end has a bifurcation that generates two processes; the more dorsal one contacts the metapterygoid via a piece of cartilage, and the ventral one extends further posteriorly and contacts the preopercle ventrally.

#### 3.8.3 Hyomandibular

The hyomandibular is formed by a perichondral ossification that begins in the hyosymplectic cartilage, surrounding the hyomandibular foramen at 4.5 mm NL with a fixed presence at 5.4 mm SL. Initially, at 5.1 mm SL, the ossification replaces the cartilage in the ventral and dorsal margins of the hyomandibular foramen (Figure 9a). By 6.8 mm SL, a thin lamellar expansion appears in the lateral anterior portion of the hyomandibular (Figure 9b). At this point, the bone covers only the dorsal portion of the hyosymplectic cartilage, with the exception of its dorsal tip, which contacts the neurocranium. At 8.6 mm SL, the hyomandibular ossification has grown ventrally, almost reaching the interhyal and has covered the hyomandibular foramen. At this stage, the anterior lamellar expansion of the hyomandibular reaches the anterior border of the foramen for the passage of the afferent pseudobranch artery (Figure 9c). In adults, the hyomandibular is roughly rectangular; the ventral end contacts the metapterygoid and symplectic while the dorsal remains cartilaginous and articulates with the sphenotic and pterotic.

#### 3.8.4 Metapterygoid

The metapterygoid begins to develop at 5.1 mm SL in the posterodorsal portion of palatoquadrate cartilage (fixed presence from 6 mm SL). The bone, at 6.8 mm SL, replaces the cartilage of the dorsal margin of the fenestra metapterygoid‐quadrate (Figure 9b). At 7.5 mm SL it reaches the endopterygoid anteriorly and the anterior lamellar expansion of the hyomandibular posteriorly. At this stage, a posterior projection of palatoquadrate cartilage is present, which will form the foramen for passage of the afferent pseudobranchial artery. The ossification of the metapterygoid progresses along this projection. From the dorsal portion anterior to the hyomandibular starts a projection with a triangular shape (8.5 mm SL). In adult specimens, the anterior region of the metapterygoid overlaps the posterior end of the ectopterygoid, posteriorly overlaps the anterior margin of the hyomandibular and borders the foramen of the afferent pseudobranch artery.

#### 3.8.5 Ectopterygoid

The ectopterygoid begins at 5.0 mm SL as a thin split of bone along the outer margin of the pterygoid process and fixes its presence at 6 mm SL (Figure 9b). The thin line progresses anteroposteriorly, and at 9.2 mm SL, the bone is already more than half the length of the pterygoid process, with its posterior tip almost reaching the quadrate (Figure 9c). In adults, the shape does not change considerably; the bone remains a thin line contacting the autopalatine anteriorly and the quadrate posteriorly.

#### 3.8.6 Endopterygoid

It is a lamellar bone that starts as elongated and thin ossification present in the inner margin of the pterygoid process at 5.1 mm SL with fixed presence at 6.8 mm SL. Along the development, the bone expands anteroposteriorly and medially. At 9.1 mm SL, the bone is formed by a medially lamellar portion with a circular shape and a small portion that invades the cartilage and makes contact with the ectopterygoid (Figure 9c). In adults, the endopterygoid remains roughly circular in shape, articulated with the metapterygoid posteriorly and with the anterior part of the ectopterygoid.

#### 3.8.7 Autopalatine

The oldest specimen analyzed did not yet have an ossified autopalatine. In adult form, the autopalatine is approximately rectangular, has a small foramen in its center, the anterior end is cartilaginous, and has a small circle of cartilage positioned anteriorly (Figure 9d). Posteriorly, it is limited by the ectopterygoid and endopterygoid.

### 3.9 Opercular series (Figure 9)

Common sequence of ossification: opercle– (interopercle + subopercle + preopercle).

#### 3.9.1 Opercle

The opercle is one of the first bones to appear in the entire skeleton; it is a dermal ossification starting at the articulation with the posterdorsal margin of hyosymplectic cartilage (at 3.0 mm NL, with fixed presence at 3.1 mm NL). At 5.1 mm SL, the bone is expanded posteroventrally and has a triangular shape with the dorsal portion concave (Figure 9a). By 7.5 mm SL, the bone has grown dorsally from the concave portion, and at 9.3 mm SL, the opercle is wide, with rounded borders, and is similar in size to the vertical portion of hyosymplectic cartilage (Figure 9b,c). In adults, the articulation with the hyomandibular is strongly ossified. The anteroventral margin of the opercle is limited by the preopercle and the ventral margin is limited by the subopercle.

#### 3.9.2 Interopercle

The interopercle begins at 5.1 mm SL as a thin splint of bone located ventrally to the sympletic (fixed presence at 6.0 mm SL). Ossification progresses anteroposteriorly, acquiring an elongated shape with a posterior tip that is larger and more rounded (Figure 9b). At 9.3 mm SL, the lamellar bone does not significantly change in shape, only increases in size and makes contact with the preopercle, overlapping it (Figure 9c). In adults, the anterior end of the interopercle extends beyond the horizontal arm of the preopercle, almost contacting the retroarticular, while the posterior end has expanded vertically, becoming much wider than the anterior region of the bone.

#### 3.9.3 Subopercle

The subopercle first appears as a thin and elongate splint of bone located ventral to the hyosymplectic cartilage (at 5.1 mm SL, fixed presence at 6.0 mm SL). This dermal bone ossifies just below the opercle and acquires a roughly rectangular shape during development (Figure 9b). At 9.3 mm SL, the bone is horizontally elongated, slightly inclined dorsoventrally, with a posterior tip triangular and an anterior tip quadrate, overlapping the interopercle. This shape does not change significantly in the adult, when the bone overlaps the opercle dorsally and the interopercle anteriorly.

#### 3.9.4 Preopercle

The preopercle is the last bone of the opercular series to appear, at 5.6 mm SL with a fixed presence at 6.0 mm SL. The preopercle ossification starts as a thin split of bone near the ventral margin of hyosymplectic cartilage, anterior to interhyal and dorsal to interopercle. The bone expands anteriorly and posteriorly, bordering the ventral margin of hyosymplectic cartilage. At 6.8 mm SL, a thin line of ossification appears above the dorsal margin of the lamellar preopercle, accompanied by two small pores (Figure 9b). The bone subsequently increases in size and acquires an inverted “L” shape, with the anterior tip connected to the sympletic and the posterodorsal tip almost reaching the articulation between the opercle and the hyomandibular (Figure 9c). In adults, the shape is similar to that described previously, but the posterodorsal end is formed by the preopercular canal that runs through the bone from that point to its anteroventral end.

### 3.10 Hyoid arch (Figure 11)

**FIGURE 11:**
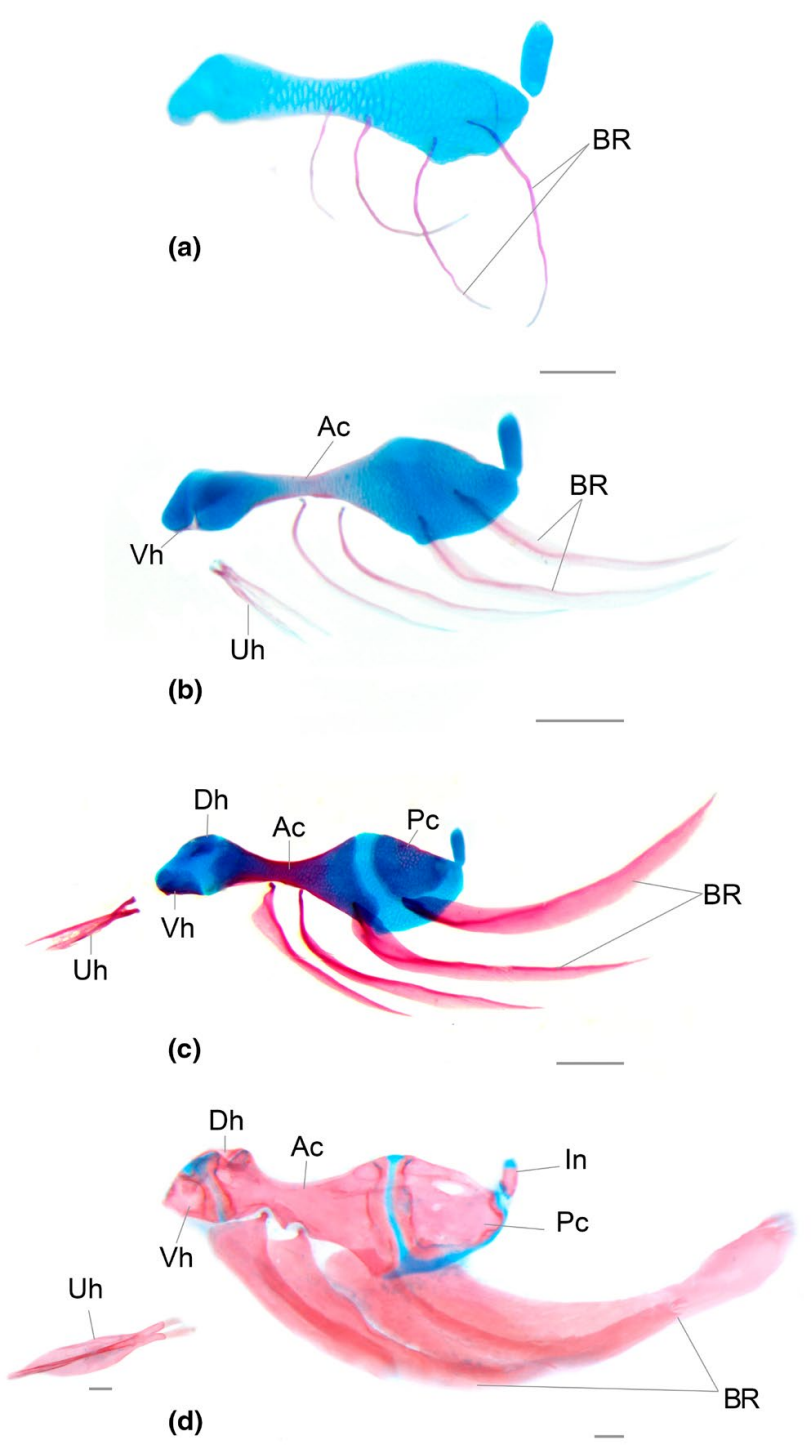
Hyoid arch of *Inpaichthys kerri*. (a) 4.3 mm NL. (b) 8.0 mm SL. (c) 9.3 mm SL. (d) 28.0 mm SL. Ac, anterior ceratohyal; BR, branchiostegal rays; Dh, dorsal hypoyal; In, interhyal; Pc, posterior ceratohyal; Uh, urohyal; Vh, ventral hypoyal. Scale bar 0.2 mm. *urohyal displaced anteriorly in C and D.

Common sequence of ossification: branchiostegal rays – anterior ceratohyal – (urohyal + ventral hypohyal) – posterior ceratohyal – (dorsal hypohyal + basihyal) – interhyal.

#### 3.10.1 Branchiostegal rays

The branchiostegal rays are the first elements of the hyoid arch to ossify, in a posterior–anterior direction. The posterior most branchiostegal ray, or the fourth one, ossifies first at 3.4 mm NL, associated with the posteroventral portion of the ceratohyal cartilage. It is followed by the third branchiostegal ray, which ossifies at 3.9 mm NL, and then by the second and first ones, which ossify simultaneously at 4.3 mm NL. Initially, all branchiostegal rays are very thin and curved (4.3 mm NL) (Figure 11a). Later, they are thicker, elongated bones, slightly curved (9.3 mm SL). In adults, the rays are longer and wider, with the anterior portion slightly wider. The first and second rays are connected to the anterior portion of the anterior ceratohyal by means of small dorsal projections, while the third and fourth rays have a straight dorsal end and are connected to the posterior end of the anterior ceratohyal and the posterior ceratohyal, respectively.

#### 3.10.2 Anterior ceratohyal

The anterior ceratohyal starts to ossify in the middle of ceratohyal cartilage, where it is thinner, at 4.0 mm SL, with fixed presence at 4.3 mm SL (Figure 11b). Ossification progresses anteriorly and posteriorly, perichondrally, then progresses by replacing the center of the ceratohyal cartilage. By 9.1 mm SL, ossification of the posterior portion of the anterior ceratohyal reaches the anterior tip of the third branchiostegal ray. At the same stage, it almost reaches the ventral and dorsal hypohyal anteriorly. In adults, the anterior ceratohyal articulates with the dorsal and ventral hypohyal anteriorly and with the posterior ceratohyal posteriorly. Its ventral margin has two notches for connection with the first two branchiostegal rays. The longitudinal canal for the passage of the hyoid artery emerges in the posteriormost region of the bone and extends to the foramen of the posterior ceratohyal.

#### 3.10.3 Urohyal

The urohyal appears for the first time at 4.6 mm SL, ventromedial to the hyoid arch (fixed presence at 5.0 mm SL). Ossification begins as a horizontal splint of bone. At 8.0 mm SL, the bone is bifurcated anteriorly and posteriorly, forming an “X” shape, with the anterior arms shorter than the posterior ones (Figure 11b). The anterior arms retain a small bifurcation in the adults, but the posterior ones have well‐developed lamella in between, roughly triangular, with two lateral flaps.

#### 3.10.4 Ventral hypohyal

The ossification starts in the anteroventral tip of ceratohyal cartilage at 4.3 mm NL, with fixed presence at 5.0 mm SL. The ventral hypohyal has grown posteriorly toward the anterior ceratohyal and dorsally toward the dorsal hypohyal. In adult specimens, the ventral hypohyal is well‐developed and triangular in shape. It articulates with the dorsal hypohyal dorsally and with the anterior ceratohyal posteriorly sinchondrally.

#### 3.10.5 Posterior ceratohyal

It begins to ossify in the posterodorsal portion of ceratohyal cartilage at 4.3 mm SL (fixed presence at 5.0 mm SL). At 9.3 mm SL, ossification progresses ventrally toward the third branchiostegal and has a semicircular shape, covering a large part of the posterior region of the ceratohyal cartilage (Figure 11c). In adults, the ventral and anterior margins of the posterior ceratohyal are surrounded by cartilage; anteriorly, it is limited by the anterior ceratohyal and posteriorly, it connects with the interhyal. In its posteriordorsal portion, there is a small foramen for the passage of the hyoid artery.

#### 3.10.6 Dorsal hypohyal

The dorsal hypohyal ossifies in the anterior dorsal portion of the ceratohyal cartilage at 5.1 mm SL, with a fixed presence at 6.0 mm SL. At 9.0 mm SL, the bone is expanded posteriorly toward the anterior ceratohyal and ventrally toward the ventral hypohyal. In adults, it is well ossified and has two arms that connect to the anterior ceratohyal and ventral hypohyal, forming a small foramen.

#### 3.10.7 Basihyal

**FIGURE 12:**
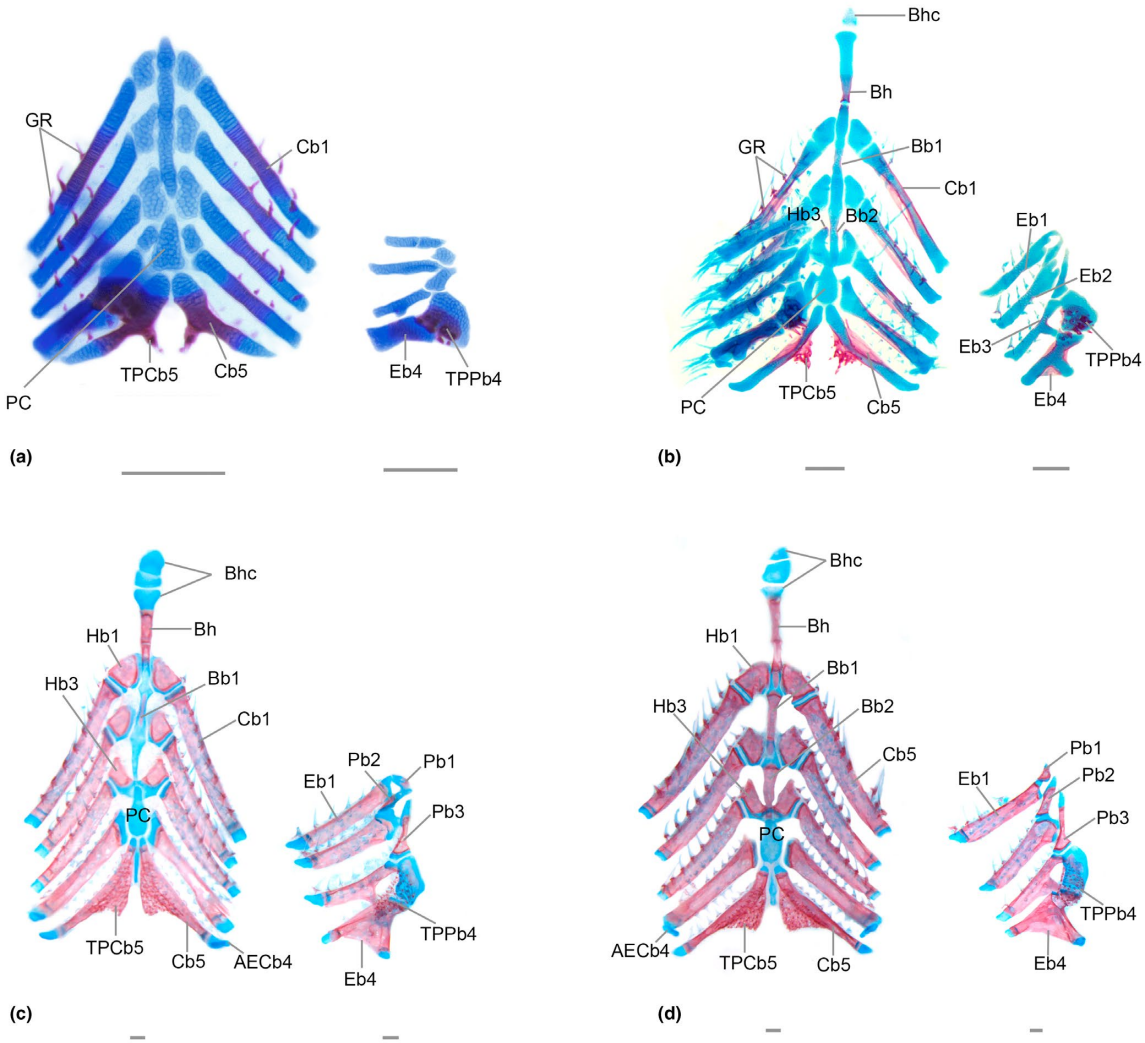
Branchial arches of *Inpaichthys kerri*. (a) 4.5 mm NL. (b) 9.3 mm SL. (c) 26.0 mm SL. (d) 28.0 mm SL. AECb4, accessory element of ceratobranchial 4; Bb1‐2, basibranchials; Bh, basihyal; Bhc, basihyal cartilage; Cb1‐5, ceratobranchials; Eb1‐4, epibranchials; GR, gill rakers; Hb1‐3, hypobranchial; Pb1‐3, pharyngobranchials; PC, posterior copula; TPCb5, tooth plate of ceratobranchial 5; TPPb4, tooth plate of pharyngobranchial 4. Scale bar 0.2 mm. *AECb4 is dislocated in C.

Appears as chondral ossification in the posterior tip of the basihyal cartilage (at 5 mm SL, with consistent presence at 6 mm SL). Ossification progresses anteriorly. At 9.3 mm SL, the bone replaced approximately half of the cartilage and has an elongated and narrow shape. In adults, the basihyal is roughly rectangular in shape, elongated, and narrow. The anterior tip of the basihyal remains cartilaginous and is limited anteriorly by two aligned autogenous cartilage blocks (Figure 12c,d). Posteriorly, the basihyal contacts the basibranchial 1.

#### 3.10.8 Interhyal

Interhyal could only be observed in the adults, where it is grain‐shaped with cartilaginous ends (Figure 11d), strongly connected to the posterior ceratohyal anteriorly and the symplectic posteriorly.

### 3.11 Branchial skeleton (Figure 12)

Common sequence of ossification: Tooth plate PB4 – Tooth plate CB5 – Ceratobranchial 5 – (Ceratobranchial 1–4) – Gill rakers – Epibranchial 4 – Epibranchial 3 – Epibranchial 2 + Basibranchial 1–3 – Epibranchial 1 – Pharingobranchial 1–3 – Hypobranchial 1–3.

#### 3.11.1 Pharyngeal jaws

The pharyngeal jaws are composed of the tooth plate associated with the pharyngobranchial 4 (upper pharyngeal jaw) and the ceratobranchial 5 (lower pharyngeal jaw), both of which appear early in development. The first ossification of the tooth plate of pharyngobranchial 4 occurs at 2.6 mm NL with fixed presence at 3.0 mm NL onward. At this stage, the tooth plate is composed of a single conical tooth oriented posteroventrally (Figure 10). At 3.2 mm NL a second, smaller, tooth appears. The number of teeth continues to increase, and at 4.5 mm NL, five teeth can be observed associated with the pharyngobranchial 4 cartilage (Figure 12a). As the tooth plate associated with pharyngobranchial 4 cartilage develops, it expands to reach a small portion of epibranchial 4. By 9.3 mm SL, it has more than 10 small and conical teeth. At 3.5 mm NL, the tooth plate of ceratobranchial 5 ossifies as one large conical tooth directed posteriorly, connected to the cartilage of ceratobranchial 5. At 6.0 mm SL, another tooth appears. By 9.0 mm SL, the number of teeth varies between 4 and 6. At this stage, the tooth plate associated with ceratobranchial 5 is narrow, while that of pharyngobranchial 4 is more rounded. In adult specimens, both dentigerous plates have several small and conical teeth; the one associated with the cartilage of pharyngobranchial 4 is approximately rectangular in shape and covers a small part of the epibranchial 4, while the one associated with ceratobranchial 5 is triangular in shape and is fully developed.

#### 3.11.2 Gill rakers

The first gill rakers to appear are those from ceratobranchials, with 3–4 rakes distributed anteriorly at the middle portion of all ceratobranchials at 4.5 mm NL, with fixed presence at 5.1 mm SL (Figure 12a). At 5.1 mm SL, there are six gill rakers anteriorly at each ceratobranchial, covering its entire extent. At 9.3 mm SL, additional gill rakers can be observed at the ceratobranchials, a total of eight to nine. At this stage, gill rakers are absent at the epibranchials and hypobranchials. In adults, the ceratobranchials and the epibranchials present two series of gill rakers, one at their anterior edge and another at their posterior edge, while the hypobranchials present only one row each. The ceratobranchials and the epibranchials have 8–10 and 4–6 gill rakers in each row, respectively, while the hypobranchials have 1–2 gill rakers in one row. The pharyngobranchials lack gill rakers. All gill rakers are ossified only at the base, except those of the posterior row of ceratobranchial 3 and 4, which are not ossified.

#### 3.11.3 Ceratobranchials

All five ceratobranchials exhibit a pattern of ossification that begins perichondrally in the middle portion of ceratobranchial cartilages and expands anteriorly and posteriorly. At 4.5 mm NL, all ceratobranchials start to ossify simultaneously. The fixed presence of ceratobranchial 5 is at 4.5 mm SL, whereas those of the remaining ceratobranchials are at 5.0 mm SL. At 9.1 mm SL, the medial portion of the cartilage is entirely replaced by bone, with only anterior and posterior cartilaginous tips. In adults, the ceratobranchials retain only their cartilaginous tips.

#### 3.11.4 Epibranchials

The epibranchials ossify in a posterior–anterior direction. The first sign of ossification appears at 5.4 mm SL in epibranchial 4, and the ossification pattern occurs similarly to that of the ceratobranchials, in which ossification progresses from the middle portion of the cartilage to its extremities. This ossification pattern is consistent for all epibranchials. At 5.5 mm SL, the epibranchial 3 cartilage starts to ossify, with fixed presence in the same length. The epibranchial 2 appears at 6.8 mm SL with fixed presence at 8.3 mm SL. The epibranchial 1 appears at 7.8 mm SL with fixed presence at 9.0 mm SL. In adults, the epibranchials are well‐developed; the first and second are thin and narrow, the third has a small uncinate process, and epibranchial 4 has a large uncinate process and is partially overlapped by the dentigerous plate of pharyngobranchial 4.

#### 3.11.5 Hypobranchials

At 4.5 mm, *Inpaichthys kerri* has four autogenous hypobranchial cartilages. Hypobranchials 1–3 begin to ossify at 6.9 mm SL while hypobranchial 4 remains cartilaginous. Ossification starts from the dorsal end of the hypobranchial 1–3 cartilages and extends posteriorly. The fixed presence of hypobranchials is assumed in the adults, as their presence is variable among larval individuals analyzed (up to 9.3 mm SL). At 6.3 mm CP, the autogenous cartilage of the hypobranchial 4 begins to fuse with the ceratobranchial 4. In the adults, there are three ossified ceratobranchials (1–3); the hypobranchial 4 remains cartilaginous and fused to the medial cartilaginous tip of ceratobranchial 4. Only hypobranchials 1–3 have gill rakers.

#### 3.11.6 Basibranchials

The basibranchials ossify in an anterior–posterior direction. Basibranchial 1 ossifies at 5.2 mm SL, basibranchial 2 at 5.6 mm SL and basibranchial 3 at 6.8 mm SL, all with fixed presence at 8.3 mm SL. Ossification of these elements begins at their posterior ends and continues anteriorly. In adults, the basibranchials are narrow and elongated and are intercalated with portions of cartilage remaining from the anterior copula. Basibranchial 4 is not ossified.

#### 3.11.7 Pharyngobranchials

All pharyngobranchials start to ossify at the middle of each pharyngobranchial cartilage, then ossification progresses to the tip of the bones. The first indication of ossification is observed at 7.8 mm SL in the pharyngobranchial 3, but it is not possible to discern the fixed presence. The oldest specimen (9.3 mm SL) analyzed showed no signs of ossification in pharyngobranchials 2 and 1. In adults, only pharyngobranchials 1–3 are ossified but still retain cartilaginous tips (Figure 12d). Pharyngobranchial 4 remains completely cartilaginous, supporting the dentigerous plate.

## 4 DISCUSSION

### 4.1 Paedomorphic features in the *Inpaichthys*

Paedomorphic features, or reductive characters, are consequences of ontogenetic truncations, in which the adult morphology is comparable to the morphology of juveniles of its ancestral or relatives (Gould, 1977). The cranial skeleton of adults of *Inpaichthys kerri* shows some notable reductive characters, which are the absence of the supraorbital bone, an incomplete infraorbital series, an incomplete supraorbital sensory canal, and an incomplete hyoid artery canal, which are discussed below. In addition, *I. kerri* has few branched pelvic fin rays and an incomplete lateral line, characters also considered paedomorphic for the group (Marinho et al., 2024). The ontogenetic truncation of these structures is associated with a process named terminal deletion, where structures that appear late in the development of large relative species are the first to be lost in small species (Britz & Conway, 2009; Hanken & Wake, 1993; Weitzman & Vari, 1988). The subsequent discussion will address these paedomorphic characteristics observed on the cranium of *I. kerri*, with consideration given to its ontogenetic trajectory and phylogenetic position.

#### 4.1.1 Orbital series

The orbital series of the adult specimens of *Inpaichthys kerri* is incomplete, lacking the supraorbital and infraorbital 6. The orbital bones are one of the last structures to appear in the development of other Characiformes (Marinho, 2022; Mattox et al., 2014; Walter, 2012). In scenarios of body size reduction, these bones are the most susceptible to be lost via terminal deletion.

The supraorbital is the last bone of the entire skeleton to appear in the development of *Salminus brasiliensis* (Bryconidae), a large Characiformes (Mattox et al., 2014). On the other hand, small species of the former Characidae family (now split into Spintherobolidae, Stevardiidae, Characidae, and Acestrorhamphidae by Melo et al., 2024) share the apomorphic absence of the supraorbital (Mirande, 2010, 2019). The loss of the supraorbital is associated with a basal ontogenetic truncation event that led to the loss of this bone in all representatives of these families (Azevedo, 2010; Marinho, 2017, 2022).

The loss of infraorbital bones is shared with the other two species of the genus. According to Ferreira et al. (2024), *Inpaichthys nambiquara* has infraorbital 4 “simplified” and 5 and 6 absent, while *I. parauapiranga* lacks infraorbitals 4, 5, and 6. In addition, *Inpaichthys* species lack the supraorbital, as do other Acestrorhamphidae species. Observing the development of the infraorbitals of Characiformes, they generally seem to follow an anterior–posterior direction in the order of appearance (Marinho, 2022, Mattox et al., 2014, Walter, 2012). In *Salminus brasiliensis*, for example, the order of the infraorbital bones is as follows: antorbital – infraorbital 1 – infraorbital 2 – infraorbital 3 – infraorbital 4 – infraorbital 5 – infraorbital 6 (Mattox et al., 2014). This order of appearance leaves the posterior infraorbitals more susceptible to bone loss and simplification in cases of ontogenetic truncation, considering terminal deletion of late developmental structures. The loss of the posteriormost infraorbital bones due to ontogenetic truncation may explain the absence of these bones in *I. kerri*, its congeners, and several other small Characiform lineages, as discussed by Marinho (2022) and Marinho et al. (2024).

#### 4.1.2 Parietal branch of the supraorbital sensory canal

In Characiformes, the supraorbital sensory canal is associated with the nasal, frontal, and parietal bones and is composed of seven canal segments and eight tubules on the dorsal surface of the skull (Pastana et al., 2019). In *Inpaichthys kerri*, the bony canal around the supraorbital sensory canal begins to ossify at 9.3 mm SL in the frontal bone, as a small bony lamella above the orbit. This ossification progresses anteriorly to the nasal tube and posteriorly to the posterior portion of the frontal. In adult specimens, the posterior portion of this canal is formed only by the SO7 tubule, a segment restricted to the frontal bone; the parietal branch or SO8 tubule is absent.

Ontogenetic truncation may be one of the causes of the loss of lateral sensory canal segments according to Pastana et al. (2019). When observing the development of *Makunaima pittieri*, a species that has a completely formed supraorbital canal, the development of the posterior portion of the supraorbital canal is at very late developmental stages. The ossification of the supraorbital canal in *M. pittieri* also starts at the frontal bone, as in *Inpaichthys kerri*, and its posterior portion, the parietal branch, only ossifies in individuals larger than 15.4 mm SL, which already have almost all the bones of the skeleton present (Marinho, 2022). In a scenario of ontogenetic truncation, the posterior part of the supraorbital canal, which is delayed in development, is the most susceptible to being lost. Notably, several lineages of small Characiformes, Spintherobolidae, Stevardiidae, Characidae, and Acestrorhamphidae share some degree of loss or simplification of the supraorbital canal, discussed by Marinho et al. (2024). The species of the genus *Inpaichthys* also share this loss of the posterior branch of the supraorbital canal, demonstrating that this event may also be associated with ontogenetic truncations.

#### 4.1.3 Hyoid artery canal on anterior ceratohyal

In Characiformes, a segment of the hyoid artery runs from the posterior ceratohyal to the dorsal hypohyal through a canal. In most lineages of small Characiformes belonging to the former Characidae, that is, in the families Spintherobolidae, Stevardiidae, Characidae, and Acestrorhamphidae, this artery enters the posterior ceratohyal through a canal, traveling along the entire horizontal length of the anterior ceratohyal until it reaches the dorsal hypohyal (Mirande, 2010, 2019; Mattox et al., 2014). In these species, the anterior ceratohyal canal runs along the entire bone, from its posterior to anterior margin. In *Inpaichthys kerri*, we observed a condition also present in the families Spintherobolidae, Stevardiidae, Characidae, and Acestrorhamphidae: the extension of the anterior ceratohyal canal is restricted to the posterodorsal region of the bone (Figure 9(d)) (Mirande et al. 2010, 2019). In these species, the hyoid artery passes through the anterior ceratohyal but exits through a pore on the posterodorsal margin of the bone and returns through an opening between the anterior ceratohyal and dorsal hypohyal (Mirande, 2010).

Observing the development of *Salminus brasiliensis*, a large species of Bryconidae, Mattox et al. (2014) pointed out that the short length of this canal in the anterior ceratohyal, observed in former Characidae, may be the result of ontogenetic truncation. In *S. brasiliensis*, this canal begins to form in the posterior region of the anterior ceratohyal and progresses to the anterior margin of the bone in later stages (Mattox et al., 2014). In *Inpaichthys kerri*, it does not reach the anterior margin of the anterior ceratohyal and is limited to the posterior region of the bone (Figure 9d), indicating a truncation in the development of the canal.

#### 4.1.4 Phylogenetic implications

**FIGURE 13:**
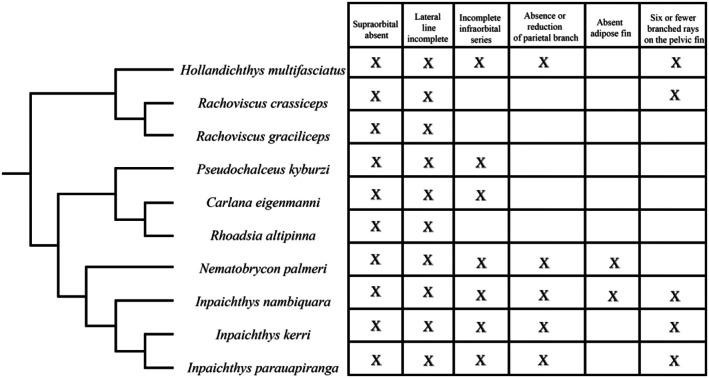
Cladogram adapted from Ferreira et al. (2024) with table indicating paedomorphic features of each species in the clade.

When analyzing such paedomorphic characters in a phylogenetic framework, the cranial paedomorphic characters found in *Inpaichthys kerri* seem to be a trend for the clade to which it belongs. In the phylogenetic analysis of the genus *Inpaichthys* carried out by Ferreira et al. (2024), the authors recovered *Hasemania nambiquara* as a species belonging to the genus *Inpaichthys* and included a new species described by them in the genus, *I. parauapiranga*. In this analysis, the genus *Inpaichthys* is sister to *Nematobrycon palmeri* and this group is nested in a clade along with the following species: *Hollandichthys multifasciatus*, *Rachoviscus crassiceps*, *R. graciliceps*, *Pseudochalceus kyburzi*, *Carlana eigenmanni*, and *Rhoadsia altipinna*. Compilation of reductive characters coded for the aforementioned species by Ferreira et al. (2024) (Figure 13) showed the same paedomorphic features found in *I. kerri* are also present in other species of the clade to which it belongs (Figure 13).

The absence of supraorbital bone, incomplete lateral line, and the incomplete infraorbital series were the most common reductive characteristics among them. Incompleteness of the lateral line is a frequent reductive character in miniature and small species of former characids, that is, families Spintherobolidae, Stervardiidae, Characidae, and Acestrorhamphidae sensu Melo et al. (2024) (Ferreira et al., 2024; Marinho et al., 2021; Mirande, 2019). Loss of infraorbital bones is also a feature found in all of these families (Ferreira et al., 2024; Mirande, 2019). Although loss of infraorbital bones is more expected in miniature species, that is, those not growing longer than 26 mm SL, it is also common in larger species, such as those from the genus *Inpaichthys* and other related species (Figure 13). Such a pattern is also true for the other paedomorphic features analyzed herein, which are also found in some larger species.

The clade composed of *Inpaichthys* species and *Nematobrycon palmeri* seems to have undergone further processes of terminal deletion, which occurred parallel in *Hollandichthys multifasciatus*. Besides exhibiting the plesiomorphic absence of supraorbital and incomplete lateral line, they all lost the parietal branch of the supraorbital canal. Furthermore, species of *Inpaichthys* have also lost pelvic fin rays. Regarding adipose fin loss, there are two evolutionary interpretations: (1) the common ancestor of the clade formed by *Nematobrycon* and *Inpaichthys* has lost the adipose fin, but the common ancestor of *I. kerri* and *I. nambiquara* regained it, or (2) *I. kerri* and *I. nambiquara* lost the adipose fin independently. It is well known that many small species of the former characid (sensu Mirande, 2019) lack the adipose fin due to developmental truncation (Marinho, 2017), and it can even vary intraspecifically in some species (Dagosta et al., 2022; Marinho et al., 2024), evidencing how labile late‐developing traits can be. Based on our analysis, independent loss of adipose fin in *I. kerri* and *I. nambiquara* via developmental truncation would be the best evolutionary scenario. Therefore, the tendency toward ontogenetic truncations seems to be stronger in the clade formed by *Nematobrycon* and *Inpaichthys* and parallel in *H. multifasciatus*, showing how variable this phenomenon can be among lineages.

### 4.2 Other features regarding the infraorbital series of *Inpaichthys kerri*

#### 4.2.1 Sequence of ossification

All adult specimens of *Inpaichthys kerri* analyzed clearly present antorbital and infraorbitals 1–5, with fused infraorbitals 4 and 5 and some specimens with fused infraorbitals 1 + 2. This is contrary to Mirande (2019), which coded the absence of antorbital and infraorbital 4 for the species. The ossification sequence of *Inpaichthys kerri* (Figure 2) revealed the infraorbital 2 is the first bone of the infraorbital series to appear, at 9.0 mm SL. This ossification occurs at the ventral margin of the connective tissue around the orbit, posterior to the maxilla and dorsal to the anguloarticular, as a thin splinter of bone positioned horizontally (Figure 7). The precise ossification sequence of the remaining infraorbital bones could not be established, but all appear subsequently to infraorbital 2.

In *Brycon moorei, Makunaima pittieri, Bario sanctaefilomenae*, and *Salminus brasiliensis*, the antorbital is always the first ossification of the infraorbital series, also appearing as a thin splinter of bone in the connective tissue around the orbit, positioned anterior to the orbitonasalis lamina and the developing lateral ethmoid (Marinho, 2022; Mattox et al., 2014; Vandewalle et al., 2005; Walter, 2012). In *M. pittieri* and *B. moorei*, following the ossification of the antorbital, the infraorbital 1 and 2 appear simultaneously, while in *S. brasiliensis* and *B. sanctaefilomenae*, infraorbital 1 appears and then infraorbital 2 sequentially.

In Cypriniformes and Siluriformes, the most anterior bony element of the infraorbital series is named infraorbital 1 or lacrimal, and it is also the first to ossify in *Danio rerio* (Cypriniformes) and in *Nocturus gyrinus* and *Ictalurus punctatus* (Siluriformes) (Cubbage & Mabee, 1996; Kubicek, 2022). In these species, the anteriormost element of the infraorbital series ossifies in the region anterior to the orbitonasalis lamina, as does the antorbital in Characiformes, with infraorbital 2 always appearing later. The ossification pattern of the infraorbital series of *Inpaichthys kerri* apparently does not follow that of other Ostariophysi with available data, and it may be related to the morphological variation found in this bone complex discussed below.

#### 4.2.2 Fusion of infraorbital bones

As described in the results, there is a morphological variation in the condition of the infraorbitals 1 and 2 of *I. kerri*. In the adult specimens analyzed, infraorbitals 1 and 2 show three distinct conditions: (1) as autogenous ossifications (Figure 8a), (2) infraorbital 1 partially fused with infraorbital 2 (Figure 8b), or (3) infraorbitals 1 and 2 completely fused (Figure 8c). As mentioned, the first infraorbital to ossify is the infraorbital 2. Therefore, we can infer that the infraorbital 1 may ossify subsequently as a separate bone or may fuse later with infraorbital 2.

**FIGURE 14:**
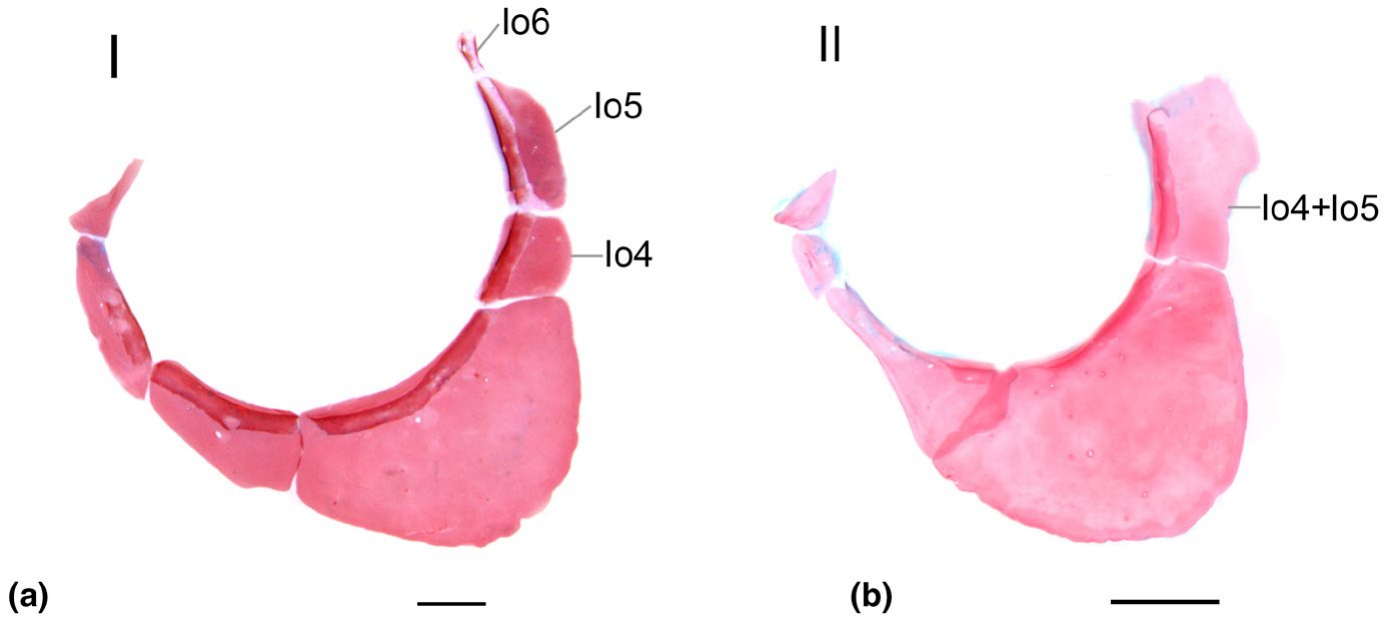
Position of infraorbitals 4 and 5 in two related species. (a) *Hyphessobrycon piabinhas*, 26 mm SL (Characidae) (Marinho et al., 2024). (b) *Inpaichthys kerri*, 28.0 mm SL. Io 4–6, infraorbitals. Scale bar 1.0 mm.

Our analyzed specimens also showed fusion in the infraorbitals 4 and 5 in *Inpaichthys kerri*, but this condition does not seem to vary. It was not possible to observe the development of infraorbitals 4 and 5, but their form and position correspond to autogenous infraorbitals 4 and 5 of other species of Characiformes (Figure 14).

The other two species of *Inpaichthys* do not exhibit infraorbital fusions. According to Ferreira et al. (2024), *I. nambiquara* has autogenous infraorbitals 1, 2, and 3 and lacks infraorbitals 4–6, whereas *I. parauapiranga* also has autogenous infraorbitals 1, 2, and 3, presents a rudimentary infraorbital 4, and lacks infraorbitals 5 and 6. Fusion of infraorbital bones is reported in the literature for a few other species belonging to the former Characidae (Acestrorhamphidae, Spintherobolidae, and Stevardiidae): *Astyanax rupestris*, *A. lorien* (Zanata et al., 2018); *A. brucutu* (Zanata et al., 2017); *A. jacobinae*, *A. epiagos* (Zanata & Camelier, 2008); *Knodus nuptialis* (Menezes & Marinho, 2019), *Myxiops aphos* (Zanata & Akama, 2004), and species of the genus *Spintherobolus* (Weitzman & Malabarba, 1999). The condition of fusion of infraorbitals 1 and 2 present in specimens of *I. kerr*i is similar to that of *Myxiops aphos* (see Figure 4a in Zanata & Akama, 2004, p. 49) and *Astyanax rupestris* (Zanata et al., 2018). The fusion of infraorbitals 4 and 5 is similar to that described for *Astyanax brucutu* (Zanata et al., 2017). All of them present intraspecific variations in these characters. *Myxiops aphos*, for example, the same specimen exhibits infraorbitals 1 and 2 and infraorbitals 3, 4, and 5 fused on the left side of the specimens, while the only infraorbitals 3 and 4 are fused on the right side.

## 5 CONCLUSION

The description of the development of the cranium of *Inpaichthys kerri* is only the sixth work on skeletogenesis done for Characiformes. Knowing the cranial bone development and ossification sequence of this species is an important contribution to the ontogenetic knowledge of Neotropical fish. We provide significant discussions about paedomorphic characters present in *I. kerri* that are shared with its cogenera and closely related species. Paedomorphosis has important morphological consequences for the evolution of broad lineages, such as the Acestrorhamphidae family, which need to be identified and discussed in the light of phylogeny. In addition, we address previously unreported morphological features of the infraorbital series of *I. kerri* related to its development and adult form. The data obtained in this study will serve as the basis for future comparative research on skeletal ontogeny in bony fishes.

## AUTHOR CONTRIBUTIONS

Yasmim De Santana Santos contributed to conceptualization, investigation, photography, and editing, writing – original draft, writing – review and editing, methodology, and data curation. Manoela M. F. Marinho contributed to conceptualization, writing – review and editing, methodology, data curation, and supervision.

## Data Availability

Data sharing not applicable to this article as no datasets were generated or analysed during the current study.
